# Genetic variants linked to myopic macular degeneration in persons with high myopia: CREAM Consortium

**DOI:** 10.1371/journal.pone.0220143

**Published:** 2019-08-15

**Authors:** Yee-Ling Wong, Pirro Hysi, Gemmy Cheung, Milly Tedja, Quan V. Hoang, Stuart W. J. Tompson, Kristina N. Whisenhunt, Virginie Verhoeven, Wanting Zhao, Moritz Hess, Chee-Wai Wong, Annette Kifley, Yoshikatsu Hosoda, Annechien E. G. Haarman, Susanne Hopf, Panagiotis Laspas, Sonoko Sensaki, Xueling Sim, Masahiro Miyake, Akitaka Tsujikawa, Ecosse Lamoureux, Kyoko Ohno-Matsui, Stefan Nickels, Paul Mitchell, Tien-Yin Wong, Jie Jin Wang, Christopher J. Hammond, Veluchamy A. Barathi, Ching-Yu Cheng, Kenji Yamashiro, Terri L. Young, Caroline C. W. Klaver, Seang-Mei Saw, The Consortium of Refractive Error, Myopia (CREAM)

**Affiliations:** 1 Singapore Eye Research Institute, Singapore National Eye Centre, Singapore, Singapore; 2 Saw Swee Hock School of Public Health, National University of Singapore, Singapore, Singapore; 3 R&D Vision Sciences AMERA, Essilor International, Singapore, Singapore; 4 Section of Academic Ophthalmology, School of Life Course Sciences, King’s College London, London, United Kingdom; 5 Duke-NUS Medical School, Singapore, Singapore; 6 Yong Loo Lin School of Medicine, National University of Singapore and National University Health System, Singapore, Singapore; 7 Department of Ophthalmology, Erasmus Medical Center, Rotterdam, The Netherlands; 8 Department of Epidemiology, Erasmus Medical Center, Rotterdam, The Netherlands; 9 Department of Ophthalmology, Columbia University Medical Center, New York, NY, United States of America; 10 Department of Ophthalmology and Visual Sciences, University of Wisconsin, Madison WI, United States of America; 11 Department of Clinical Genetics, Erasmus Medical Center, Rotterdam, The Netherlands; 12 Institute for Medical Biostatistics, Epidemiology and Informatics, University Medical Center of the Johannes Gutenberg—University Mainz, Mainz, Germany; 13 Institute of Medical Biometry and Statistics, Faculty of Medicine and Medical Center—University of Freiburg, Freiburg, Germany; 14 Department of Ophthalmology, Centre for Vision Research, Westmead Institute for Medical Research, University of Sydney, Sydney, New South Wales, Australia; 15 Department of Ophthalmology and Visual Sciences, University Graduate School of Medicine, Kyoto, Japan; 16 Department of Ophthalmology, University Medical Center of the Johannes Gutenberg—University Mainz, Mainz, Germany; 17 Department of Ophthalmology and Visual Science, Tokyo Medical and Dental University, Tokyo, Japan; 18 Department of Ophthalmology, Otsu Red-Cross Hospital, Otsu, Japan; 19 Department of Ophthalmology, Radboud University Medical Center, Nijmegen, The Netherlands; Kunming Institute of Zoology, Chinese Academy of Sciences, CHINA

## Abstract

**Purpose:**

To evaluate the roles of known myopia-associated genetic variants for development of myopic macular degeneration (MMD) in individuals with high myopia (HM), using case-control studies from the Consortium of Refractive Error and Myopia (CREAM).

**Methods:**

A candidate gene approach tested 50 myopia-associated loci for association with HM and MMD, using meta-analyses of case-control studies comprising subjects of European and Asian ancestry aged 30 to 80 years from 10 studies. Fifty loci with the strongest associations with myopia were chosen from a previous published GWAS study. Highly myopic (spherical equivalent [SE] ≤ -5.0 diopters [D]) cases with MMD (N = 348), and two sets of controls were enrolled: (1) the first set included 16,275 emmetropes (SE ≤ -0.5 D); and (2) second set included 898 highly myopic subjects (SE ≤ -5.0 D) without MMD. MMD was classified based on the International photographic classification for pathologic myopia (META-PM).

**Results:**

In the first analysis, comprising highly myopic cases with MMD (N = 348) versus emmetropic controls without MMD (N = 16,275), two SNPs were significantly associated with high myopia in adults with HM and MMD: (1) rs10824518 (P = 6.20E-07) in *KCNMA1*, which is highly expressed in human retinal and scleral tissues; and (2) rs524952 (P = 2.32E-16) near *GJD2*. In the second analysis, comprising highly myopic cases with MMD (N = 348) versus highly myopic controls without MMD (N = 898), none of the SNPs studied reached Bonferroni-corrected significance.

**Conclusions:**

Of the 50 myopia-associated loci, we did not find any variant specifically associated with MMD, but the *KCNMA1* and *GJD2* loci were significantly associated with HM in highly myopic subjects with MMD, compared to emmetropes.

## Introduction

Myopia is a refractive error condition that can usually be corrected with visual aids. It may however result in significant complications, as high myopia (HM) increases the risk of myopic macular degeneration (MMD). MMD, defined as the presence of myopia-specific retinal pathology from excessive axial elongation, is characterized by structural degeneration of the retina and associated with changes in the scleral wall [1]. MMD is one of the leading causes of irreversible loss of vision and blindness worldwide [2–5]. Numerous genome-wide association studies (GWAS) have identified multiple genetic variants associated with myopia or spherical equivalent (SE) in the general population [6–12]. Several association studies [13–19] also suggested overlapping genetic risk between myopia and HM that often correlate with blinding complications [20]. Currently, only a relatively small number of loci have been associated with HM [21–26].

Several single nucleotide polymorphisms (SNPs) associated with MMD have been identified in previous GWAS analyses in Japanese populations [27]. However, only one GWAS identified a locus specific to MMD at rs11873439 in *CCDC102B*, which compared high myopes with MMD (cases) with high myopes without MMD (controls) [28]. In addition, some studies had ambiguous definitions of MMD that did not refer to a single and formal classification system, which limits comparability of findings [29, 30]. Therefore, genetic determinants of MMD require validation using the recently established International META-PM classification system for MMD [28].

For two of the most highly significant SNPs first associated with refractive error (*GJD2* and *RASGRF1*), no association with MMD was found in an ethnically-homogenous Chinese population [31]. Further international studies with ethnically diverse populations are needed to evaluate the roles of these variants in MMD.

In this study, we evaluated the roles of known myopia-associated genetic variants in HM and MMD, using case-control studies from the Consortium of Refractive Error and Myopia (CREAM).

## Methods

### Study population and design

Subjects of either European or Asian ancestry, with available genome-wide genotyping and MMD status information, from 10 different cohorts participating in the CREAM consortium (Table 1), were included in this study [7, 15]. Subjects were between 30 and 80 years of age. A previous history of cataract surgery or laser refractive procedures that could alter refraction, were criteria for exclusions from the analyses. A total of 17,521 subjects were included in this study.

**Table 1:** Characteristics of cases (high myopes with myopic macular degeneration [MMD]) versus controls in first control set (emmetropes) and second control set (high myopes without MMD).

Study, country	Ethnicity	Meta-PM classifi-cation		Cases (high myopes with MMD) [N = 348]			First control set (emmetropes without MMD) [N = 16,275]			Second control set (high myopes without MMD) [N = 898]		
			Total [N = 17,521]	N	Mean Age (SD)	Mean SE (SD)	N	Mean Age (SD)	Mean SE (SD)	N	Mean Age (SD)	Mean SE (SD)
**Subjects of European Ancestry**												
Blue Mountains Eye Study (BMES), Australia	White European	Yes	1,519	22	60.7 (6.8)	-9.8 (3.2)	1,480	63.8 (7.6)	1.2 (1.3)	17	58.7 (7.3)	-5.9 (1.1)
Rotterdam Study I (RS1), Netherlands	White European	Yes	4,340	46	70.1 (9.1)	-6.9 (3.7)	4,165	66.7 (6.6)	2.5 (1.7)	129	67.9 (10.0)	-5.6 (2.4)
Rotterdam Study II (RS2)	White European	Yes	1,650	35	68.0 (8.4)	-8.7 (3.3)	1,569	64.6 (7.4)	1.5 (1.5)	46	62.7 (5.1)	-6.1 (1.8)
Rotterdam Study III (RS3)	White European	Yes	1,668	25	56.3 (4.1)	-8.1 (3.9)	1,533	62.3 (5.6)	1.0 (1.4)	110	59.2 (5.4)	-7.1 (1.6)
Gutenberg Health Study (GHS) 1, Germany	White European	Yes	919	7	55.3 (10.7)	-13.6 (3.3)	848	52.7 (10.1)	0.0 (0.3)	64	52.0 (10.2)	-8.0 (2.6)
GHS2	White European	Yes	403	2	62.5 (10.7)	-8.5 (1.2)	366	52.4 (10.6)	0.0 (0.3)	35	47.8 (8.4)	-8.0 (1.6)
**Subtotal for Subjects of European Ancestry**			**10,499**	**137**	**64.7 (8.0)**	**-8.4 (3.5)**	**9,961**	**63.5 (7.3)**	**1.6 (1.5)**	**401**	**60.2 (8.3)**	**-6.7 (2.1)**
**Subjects of Asian Ancestry**												
Singapore Chinese Eye Study (SCES), Singapore	Chinese	Yes	1,529	38	58.1 (8.5)	-10.0 (3.6)	1,357	58.9 (8.9)	0.8 (1.0)	134	51.9 (5.8)	-6.9 (1.7)
Singapore Malay Eye Study (SiMES), Singapore	Malay	Yes	1,849	26	61.5 (11.5)	-8.8 (3.6)	1,779	58.7 (10.2)	0.7 (1.0)	44	50.4 (8.3)	-7.4 (2.3)
Singapore Indian Eye Study (SINDI), Singapore	Indian	Yes	1,725	17	57.6 (8.2)	-8.6 (3.3)	1,641	56.7 (8.8)	0.9 (1.1)	67	51.7 (6.8)	-6.9 (1.7)
Nagahama cohort study, Japan	Japanese	Yes	1,919	130	52.1 (12.0)	-8.6 (3.0)	1,537	57.2 (12.2)	0.6 (1.0)	252	43.1 (10.5)	-7.2 (1.6)
**Subtotal for Subjects of Asian Ancestry**			**7,022**	**211**	**54.8 (11.1)**	**-8.9 (3.2)**	**6,314**	**57.9 (10.1)**	**0.7 (1.0)**	**497**	**47.3 (8.8)**	**-7.1 (1.7)**

Abbreviations: MMD, myopic macular degeneration; SE, spherical equivalent; SD, standard deviation.

The prevalence of MMD is higher in Asian cohorts, therefore most cases included in the analyses were of Asian ancestry. The Singapore Epidemiology of Eye Diseases (SEED) studies, consisting of the Singapore Chinese Eye Study (SCES), the Singapore Malay Eye Study (SiMES) and the Singapore Indian Eye Study (SINDI), contributed a total of 81 cases, 4,777 controls (set 1), and 245 controls (set 2) [32, 33]. Another study consisting of individuals of Asian ancestry, namely the Nagahama Study, contributed 130 cases, 1,537 controls (set 1), and 252 controls (set 2). The Rotterdam Studies (RS), comprising the RS1, RS2 and RS3 cohorts, contributed 106 cases, 7,267 controls (set 1), and 285 controls (set 2) [34–38]. Other studies with subjects of European ancestry contributed 22 cases, 1,480 controls (set 1) and 17 controls (set 2) from the Blue Mountains Eye Study (BMES); and a total of 9 cases, 1,214 controls (set 1) and 99 controls (set 2) from the Gutenberg Health Study (GHS) 1 and GHS2 [39]. All studies were performed with the approval of their local medical ethics committee, and written informed consent was obtained from all participants in accordance with the Declaration of Helsinki. The names of the ethics committees of the individual studies are listed in the Supporting Information (S2 Text).

### Phenotypic assessment

Each subject underwent detailed ophthalmologic examination. Non-cycloplegic refraction status was determined by the use of an autorefractor and/or subjective refraction. SE of refractive error was defined as sphere plus half cylinder. Emmetropia and HM were defined as SE > -0.5 D and ≤ -5.0 D in the right eye, respectively [40].

Fundus photograph grading was performed by trained graders for all HM subjects with SE ≤ -5.0 D in the right eye. The graders from each participating study underwent training by experienced retinal specialist (K.O.M.), and all participating studies defined MMD based on the International META-PM Photographic Classification and Grading System for MMD [28]. The presence of MMD was defined and classified into Meta-PM categories. MMD was graded according to increasing severity: no macular lesions (category 0), tessellated fundus only (category 1), diffuse chorioretinal atrophy (category 2), patchy chorioretinal atrophy (category 3), and macular atrophy (category 4). Based on fundus photograph grading, the subject was considered to have MMD, if Meta-PM category 2, 3, or 4, was observed [1].

### Evaluation of the role of myopia-associated genetic variants with MMD

We used a candidate gene approach that tested 50 genetic variants for association with MMD. The 50 selected genetic variants were reported to be associated with myopia from the largest GWAS study published to date [11]. Two case-control analyses were performed to evaluate the roles of known myopia-associated genetic variants with the development of MMD in highly myopic persons.

The first analysis aimed at identifying genetic variants associated with HM in highly myopic subjects with MMD. It compared 348 highly myopic cases with MMD (SE ≤ -5.0 D in the right eye; mean SE range between -6.9 and -13.6 D) with 16,275 emmetropic controls without HM or MMD (SE ≥ -0.5 D in the right eye; mean SE range between 0.6 and 2.5 D). Of the 348 cases included, 137 (39.4%) and 211 (60.6%) cases were of European and Asian ancestries, respectively. Of the 16,275 emmetropic controls, 9,961 (61.2%) and 6,314 (38.8%) were of European and Asian ancestries, respectively.

The second analysis aimed at identifying genetic variants specifically associated with MMD. We used the same group of cases (348 high myopes with MMD) and compared them with a control set different from the previous one, that comprised 898 high myopes without signs of MMD (mean SE range between -5.6 and -8.0 D). Of the 898 highly myopic controls, 401 (44.7%) and 497 (55.3%) were of European and Asian ancestries, respectively.

Genotyping and imputation were executed as previously described [41]. Stringent quality control (QC) procedures of genotyping before imputation were applied in each study. Briefly, duplicate DNA samples, subjects with low call rate (< 95%), gender mismatch, or ethnic outliers were excluded. SNPs were excluded if they had a low genotyping call rate (> 5% missingness), a minor allele frequency (MAF) of less than 1%, or were Hardy-Weinberg disequilibrium (P < 10^−6^). After QC filtering, genomic imputation was performed using the 1000 Genomes Project data as reference panel (build 37, phase 1 release, March 2012) with Minimac [42] or IMPUTE2 [43]. SNPs with MAF ≥ 5% and imputation quality of at least 0.5 (*r*^2^ for MACH or info score for IMPUTE) were included in further analyses.

### Gene expression in human ocular tissues

Adult ocular samples were obtained from the normal eyes of an 82-year-old Caucasian female from the North Carolina Eye Bank, Winston-Salem, North Carolina, USA. Fetal ocular samples were obtained from 24-week fetal eyes by Advanced Bioscience Resources Inc., Alameda, California, USA. The adult ocular samples were stored in Qiagen RNA later within 6.5 hours of collection and shipped on ice overnight to the lab. Fetal eyes were preserved in RNA later within minutes of harvesting and shipped over night on ice. Whole globes were dissected on the arrival day. Isolated tissues were snap-frozen and stored at −80°C until RNA extraction. RNA was extracted from each tissue sample independently using the Ambion *mir*Vana total RNA extraction kit. The tissue samples were homogenized in Ambion lysis buffer with an Omni Bead Ruptor Tissue Homogenizer per protocol. Reverse transcription reactions were performed with Invitrogen SuperScript III First-Strand Synthesis kit. The expression of the identified genes was assessed by running 10 μl reactions with QIAGEN’s PCR products consisting of 1.26 μl H_2_O, 1.0 μl 10× buffer, 1.0 μl dNTPs, 0.3 μl MgCl, 2.0 μl Q-Solution, 0.06 μl taq polymerase, 1.0 μl forward primer, 1.0 μl reverse primer, and 1.5.0 μl cDNA. The reactions were run on an Eppendorf Mastercycler Pro S thermocycler with touchdown PCR ramping down 1°C per cycle from 72°C to 55°C followed by 50 cycles of 94°C for 30 s, 55°C for 30 s, and 72°C for 30 s with a final elongation of 7 min at 72°C. All primer sets were designed by Primer3 [44]. Gel electrophoresis was run on a 2% agarose gel at 70 volts for 35 minutes. The primers were run on a custom tissue panel including the Clontech Human MTC Panel I, Fetal MTC Panel I, and an ocular tissue panel.

### Statistical analysis

Logistic regression models were performed for all studies with each SNP as predictors, and MMD as a binary outcome, with adjustments for age, gender, and principal components. To avert population stratification and inflation of the results in each cohort, the ancestry of all participants was checked via a Principal Component Analysis. Individuals who were not perfectly clustering with their respective ethnic groups were removed. Meta-analyses were performed to estimate the combined effects, using inverse-variance fixed-effect meta-analyses in METAL [45]. The meta-analyses were stratified by ancestry (European or Asian ancestry). Only SNPs that were available and polymorphic in at least 8 participating studies were considered. Of the 50 SNPs, 39 and 37 were included in the first analysis (highly myopic cases with MMD versus emmetropic controls without MMD) and second analysis (highly myopic cases with MMD versus highly myopic controls without MMD), respectively. Corrections for multiple testing were performed: P_Bonferroni_ = 0.05/39 = 1.28E-03 for the first analysis and P_Bonferroni_ = 0.05/37 = 1.35E-03 for the second analysis.

## Results

The highly myopic cases with MMD (mean SE of -8.67 ± 3.3 D; N = 348) were more myopic than the highly myopic controls without MMD (mean SE of -6.89 ± 1.89 D; N = 898). The mean SE of the emmetropic controls without MMD was 1.43 ± 1.43 D (N = 16,275). Compared to the subjects of European ancestry, subjects of Asian ancestry were more myopic in the cases and two control groups (Table 1).

### (1) Evaluation of genetic variants associated with HM in highly myopic subjects with MMD

**Fig 1:**
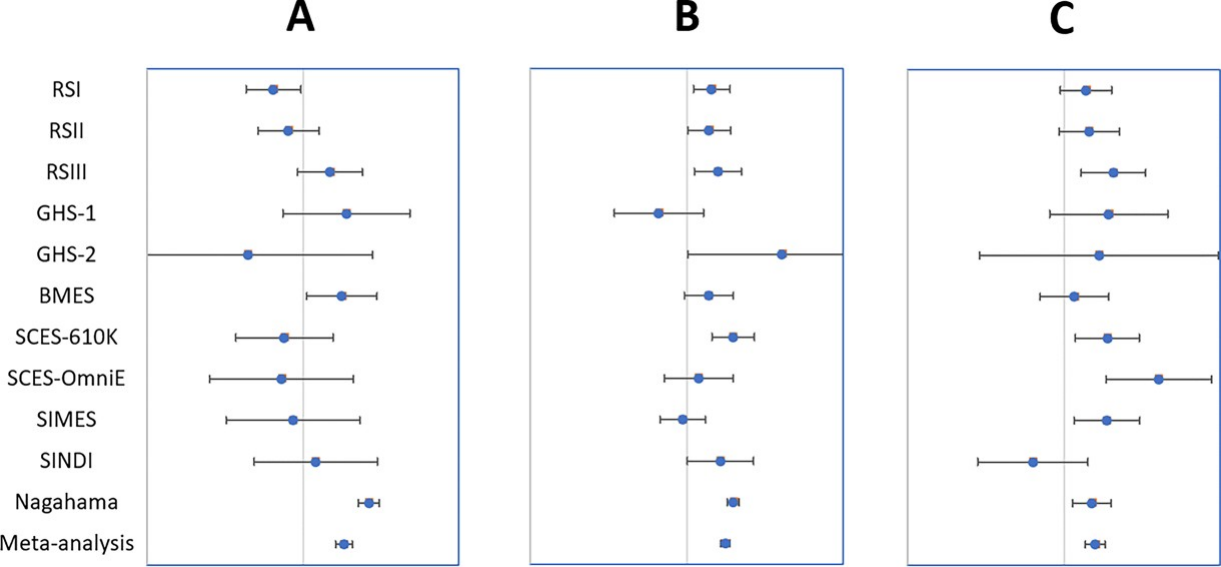
**Plot of the effect on high myopia in highly myopic subjects with myopic macular degeneration for (A) rs10824518, (B) rs524952, and (C) rs13380104 in the population cohorts in first case-control study.** For each cohort, the circle shows the β linear regression coefficient and the bars represent the standard error for the estimate. BMES, the Blue Mountains Eye Study, Australia; RS-I, the first Rotterdam Study cohort, Netherlands; RS-II, the second Rotterdam Study cohort, Netherlands; RS-III, the third Rotterdam Study cohort, Netherlands; GHS1, the first Gutenberg Health Study cohort, Germany; GHS2, the second Gutenberg Health Study cohort, Germany; SCES, the Singapore Chinese Eye Study, Singapore; SiMES, the Singapore Malay Eye Study, Singapore; SINDI, the Singapore Indian Eye Study, Singapore; Nagahama, the Nagahama Study cohort, Japan.

In the first analysis (highly myopic cases with MMD versus emmetropic controls without MMD), two SNPs were significantly associated with HM in highly myopic subjects with MMD (Table 2). rs10824518 (P = 6.20E-07; Fig 1A) maps within the *KCNMA1* gene genomic sequence and rs524952 (P = 2.32E-16; Fig 1B) about 38kbp downstream the *GJD2* gene. A third SNP, rs13380104 (P = 1.73E-03; Fig 1C), located in the last intron of the *RASGRF1* gene, was just short of our pre-defined Bonferroni corrected threshold of significance.

**Table 2:** List of the 10 SNPs most significantly associated with HM in highly myopic subjects with myopic macular degeneration (MMD) from the meta-analysis in first case-control study (cases [high myopes with MMD] versus first control set [emmetropes]).

SNP	Gene	Allele1	Allele2	Effect size	Standard Error	P-value	I^2^ (heterogenity)	X^2^ (heterogenity)	Deg of freedom	P-value (heterogenity)
rs524952	*GJD2*	a	T	0.4941	0.0602	2.32E-16	3.8	10.396	10	0.4065
rs10824518	*KCNMA1*	a	T	0.3691	0.0741	6.20E-07	49.1	19.637	10	0.03288
rs13380104	*RASGRF1*	t	C	0.2809	0.0897	1.73E-03	0	3.922	10	0.9508
rs7162310	*APH1B*	t	C	-0.227	0.0979	2.04E-02	31.1	14.506	10	0.1511
rs2908972	*SHISA6*	a	T	0.1843	0.0927	4.69E-02	0	5.675	10	0.8418
rs11606250	*LRRC4C*	a	G	0.1913	0.1031	6.36E-02	0	7.841	10	0.6444
rs4948523	*BICC1*	a	C	0.1444	0.0814	7.60E-02	0	8.966	10	0.5353
rs7968679	*PZP*	a	G	-0.1423	0.12	2.36E-01	0	9.033	10	0.529
rs1793639	*NTM*	a	G	-0.0662	0.101	5.12E-01	0	4.577	10	0.9176
rs11658305	*POLR2A/ TNFSF12/ TNFSF13*	a	C	-0.0196	0.0862	8.20E-01	27.5	13.797	10	0.1825

### (2) Evaluation of genetic variants specifically associated with MMD

In the second analysis, (highly myopic cases with MMD versus highly myopic controls without MMD), none of the SNPs reached Bonferroni-corrected significance in this model (Table 3). The highest association was observed for rs479445 (P = 2.55E-02), located downstream of the *NFIA* gene.

**Table 3:** List of the 10 SNPs most significantly associated with myopic macular degeneration (MMD) exclusively from the meta-analysis in second case-control study (cases [high myopes with MMD] versus second control set [high myopes without MMD]).

SNP	Gene	Allele1	Allele2	Effect size	Standard Error	P-value	I^2^ (heterogenity)	X^2^ (heterogenity)	Deg of freedom	P-value (heterogenity)
rs479445	*C1orf87 /NFIA*	a	T	0.2955	0.1323	2.55E-02	0	7.97	10	0.6317
rs2207136	*TFAP2B*	t	C	0.2508	0.1346	6.24E-02	0	9.133	10	0.52
rs13380104	*RASGRF1*	t	C	0.1564	0.1304	2.30E-01	0	5.808	10	0.8312
rs7744813	*KCNQ5*	a	C	-0.154	0.1391	2.68E-01	0	5.486	10	0.8564
rs2808510	*NR5A2 /ZNF281*	t	C	0.129	0.1281	3.14E-01	30	14.276	10	0.1608
rs11606250	*LRRC4C*	a	G	0.1198	0.1514	4.29E-01	0	5.223	10	0.8758
rs2799081	*PGBD1 /ZSCAN31*	t	C	0.1036	0.1374	4.51E-01	0	4.2	10	0.9379
rs2155413	*DLG2*	a	C	-0.0664	0.1276	6.03E-01	13.9	11.614	10	0.3117
rs10824518	*KCNMA1*	a	T	-0.0727	0.1486	6.25E-01	0	4.519	10	0.9209
rs4948523	*BICC1*	a	C	0.0416	0.1239	7.37E-01	0	4.918	10	0.8966

**Fig 2:**
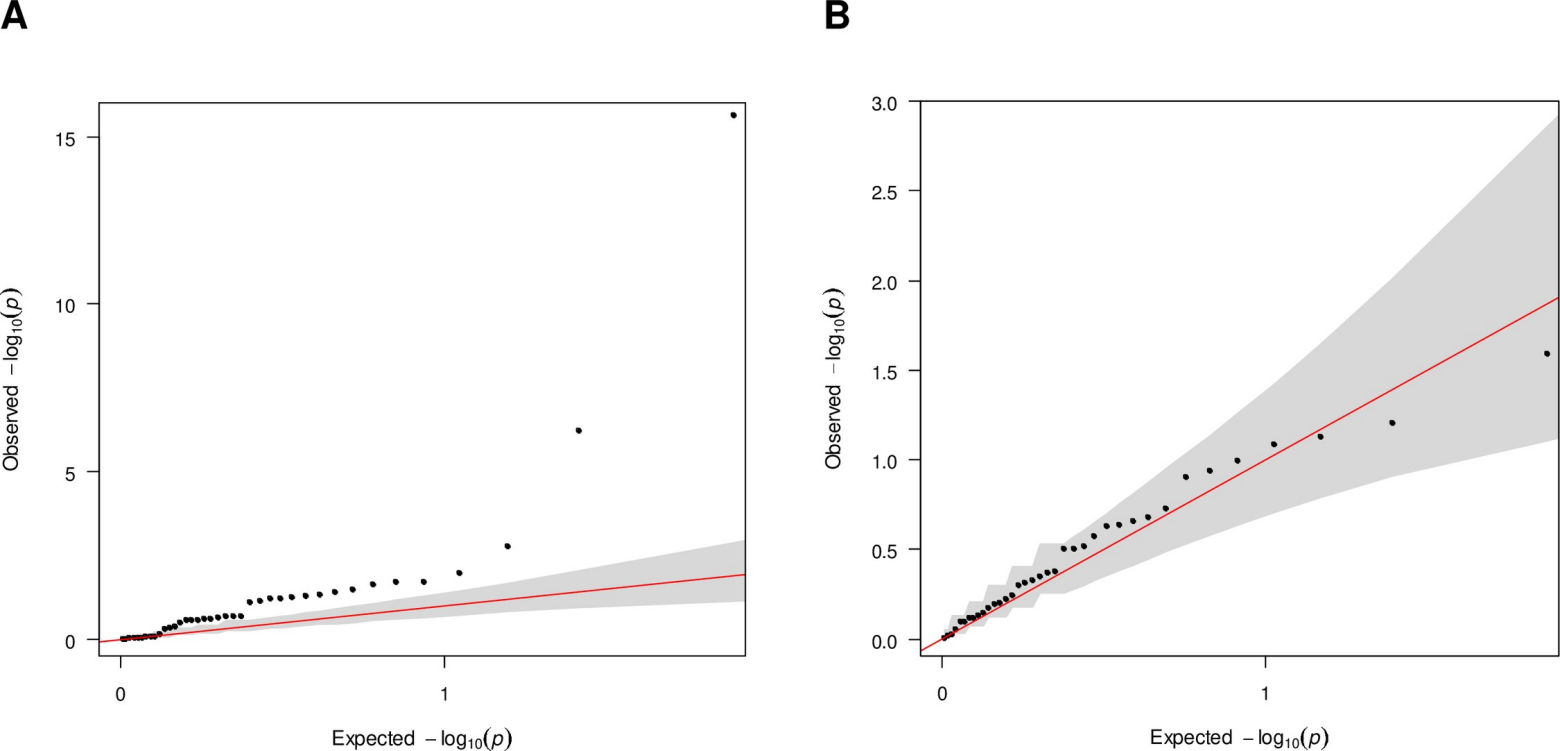
Quantile-quantile plots for the meta-analyses in first and second case-control studies. (A) Q-Q plot for association between analysed SNPs and HM in high myopes with MMD in first case-control study (highly myopic cases with MMD versus emmetropic controls without MMD); (B) Q-Q plot for association between analysed SNPs and MMD specifically in second case-control study (highly myopic cases with MMD versus highly myopic controls without MMD). Each dot represents an observed statistic (defined as–log10p) versus the corresponding expected statistic. The red line corresponds to the null distribution. The shaded areas represent the 95% confidence intervals.

To assess whether the SNPs associated with myopia had any role in HM and MMD predisposition, quantile-quantile plots of the P-values from each meta-analysis were examined (Fig 2). Associations of genetic variants for HM and MMD between highly myopic cases with MMD and emmetropic controls without MMD showed significance, beyond what would be expected under the assumption of a uniform distribution (Kolmogorov Smirnov for uniformity p = 3.87E-05), as the test statistic distribution deviated from expectations for the first analysis (Fig 2A). In contrast, after the influence of HM was removed in the second analysis, the associations of genetic variants for MMD between highly myopic cases with MMD and highly myopic controls without MMD were not significantly different from the null hypothesis of a uniform distribution (p = 0.64) (Fig 2B).

**Fig 3:**
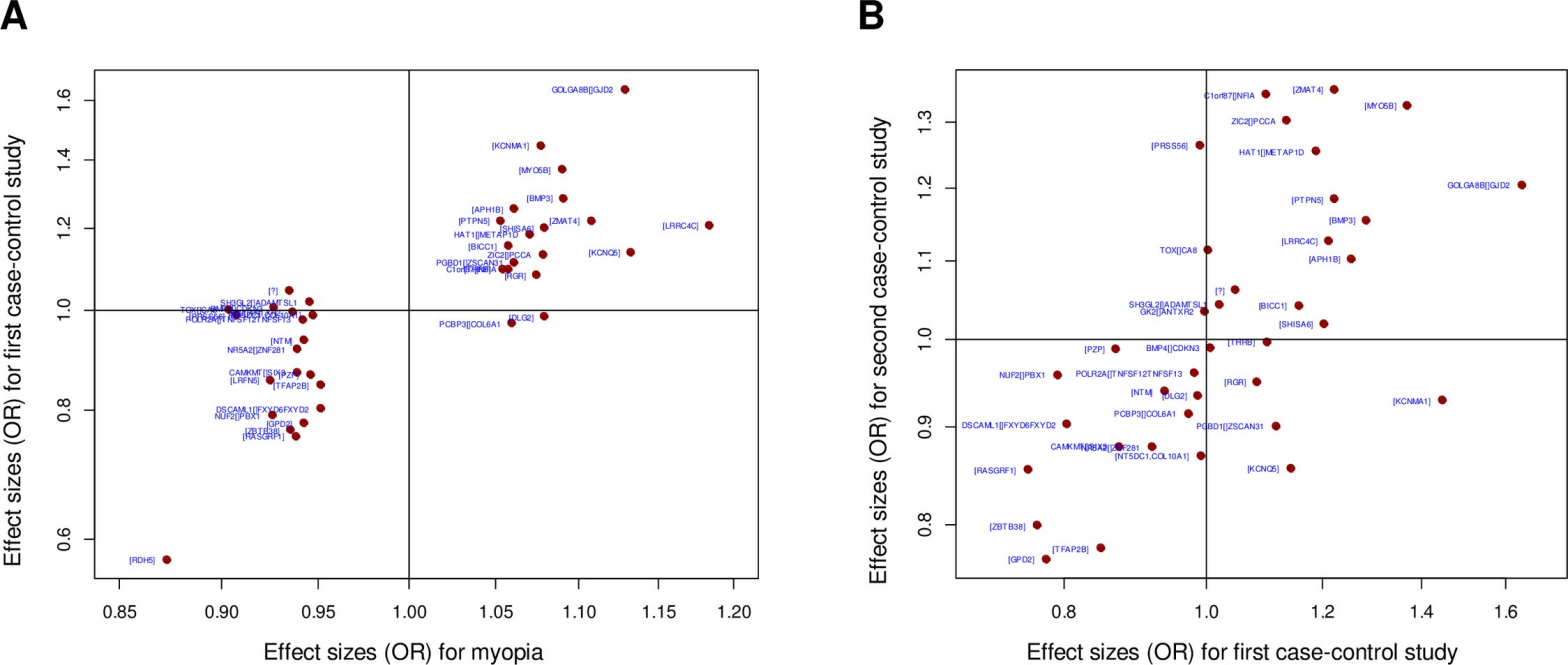
Association plots of effect sizes for the meta-analyses in first and second case-control studies. (A) Relation of effect sizes observed in original study for myopia (Pickrell et al 2016) versus that in the first case-control study (highly myopic cases with MMD versus emmetropic controls without MMD) for HM in high myopes with MMD; (B) Relation of effect sizes in first case-control study (highly myopic cases with MMD versus emmetropic controls without MMD) for development of MMD in those with HM versus that in the second case-control study (highly myopic cases with MMD versus highly myopic controls without MMD) for MMD specifically.

The effect sizes of the genetic variants reported for myopia were strongly correlated with the effect sizes of the SNPs in the first analysis (Fig 3A), reflecting the correlation (Spearman’s ρ = 0.70, p = 1.15E-07) of myopia with HM and MMD. Several genetic loci (such as *KCNMA1* and *GJD2*) displayed stronger effects over HM and MMD than SE. Alternatively, for some other loci previously associated with myopia [11], we observed weaker or no effect at all over HM and MMD (for example *DLG* and *COL61A*). There was a marginally weaker correlation of the effect sizes between the first and second case-control analysis (Spearman’s ρ = 0.59, p = 0.0001, Fig 3B), perhaps reflecting an overlap of genetic risks between HM and MMD.

### Gene expression in human ocular tissues

As the expression and role of *GJD2* and *RASGRF1* in eye and myopia development have been explored and reported previously [6–9, 15], we focused on the gene expression of *KCNMA1* in human ocular tissues. *KCNMA1* was expressed in most adult and fetal ocular tissues, including human retina, sclera, choroid or retinal pigment epithelium (RPE), and optic nerve (Table 4). In particular, *KCNMA1* was highly expressed in human retina and sclera for both fetal and adult tissues.

**Table 4:** Expression of *KCNMA1* in the various human eye tissues.

		*KCNMA1*	
	Tissue	Expression	p-value
Retina	Adult Retina	**890.32**	**<0.001**
	Adult Peripheral Retina	**551.12**	**<0.001**
	24 week Retina/RPE	**560.04**	**<0.001**
	24 week Peripheral Retina/RPE	**366.33**	**<0.001**
	12 week Retina/ RPE/Choroid	52.91	0.10
Sclera	Adult Sclera	**1013.48**	**<0.001**
	Adult Peripheral Sclera	743.71	0.14
	24 week Sclera	**317.22**	**<0.001**
	24 week Peripheral Sclera	**434.30**	**<0.001**
	12 week Sclera	**124.44**	**<0.001**
Choroid /RPE	Adult Choroid	**152.77**	**<0.001**
	Adult Peripheral Choroid	**179.41**	**<0.001**
	24 week Choroid	**218.18**	**<0.001**
	24 week Peripheral Choroid	**257.90**	**<0.001**
Optic nerve	Adult Optic nerve	**549.38**	**<0.001**
	Fetal Optic nerve	**162.67**	**<0.001**

Abbreviations: RPE, retinal pigment epithelium.

## Discussion

Using two case-control meta-analyses, this study evaluated genetic risk factors for the development of MMD in adults with HM who have MMD. We found that *KCNMA1* is linked to HM in highly myopic individuals with MMD in CREAM, a locus that had been previously identified for myopia in CREAM and 23andMe [12]. Furthermore, we replicated previously reported association on *GJD2* and *RASGRF1* in highly myopic individuals with MMD compared to emmetropic controls without MMD. However, these results were not replicated in the second case-control study that compared highly myopic cases with MMD and highly myopic controls without MMD. Since these genetic variants were not tested positive in both case-control studies, we found no evidence that any of the variants that we analysed, confers risk specific to MMD, beyond risk mediated through HM. These genes might be linked to development of MMD only in these highly myopic subjects with MMD.

Several genetic variants associated with MMD have been reported in the literature [29, 30], but few have been consistently replicated [31]. A previous GWAS identified a genetic locus associated with MMD at rs11873439 in *CCDC102B* (N = 7739; P = 1.61E-10; odds ratio [OR] of 1.46; 95% confidence interval [CI], 1.30 to 1.64). The *CCDC102B* gene protein may be linked to weakened connective tissue in retinal and choroid layers, which predisposes the eye to MMD [27]. Another GWAS analysis (N = 2,741) found an association between rs577948 in *BLID* (OR of 1.37; 95% CI, 1.21 to 1.54; P = 2.22E-07) [29], which encodes an inducer of mitochondrial cell death and apoptosis and expressed in human retina [46]. We did not find similar results to previous studies, as this could potentially be due to small sample size or the greater complexity of MMD that may have multifactorial, polygenic and environmental influences.

We have confirmed at least the *KCNMA1* locus (10q22) as a susceptibility locus for HM in persons with both HM and MMD. *KCNMA1* was identified as a susceptibility locus for SE and myopia in the wider general population in two previous large GWAS [6, 11, 12], but we observed much stronger effects and association near the high myopic end of the refraction spectrum. Encoding a large potassium voltage-sensitive conductance calcium-activated channel (MaxiK+) [47], *KCNMA1* is mainly involved in ion channel activity [48], control of smooth muscle and neuronal development [49], action potential repolarization of neurons [50], regulation of neurotransmitter release [51] and synaptic plasticity [47]. Notably, MaxiK+ channels control synaptic transmission exclusively in the rod pathway, a light-induced signalling pathway that contributes to myopia development [52]. *KCNMA1* is expressed in neurons, retinal, and RPE tissues [47, 51, 53]. MaxiK+ channels in RPE control the changes in intracellular Ca2+, in turn regulating several cell functions including dark adaptation of photoreceptor activity, differentiation and vascular endothelial growth factor (VEGF) secretion [54, 55], thereby suggesting possible involvement of *KCNMA1* in myopia-related pathologic changes, such as the initiation of choroidal neovascularization and changes in the blood-retinal barrier [56]. Validation of the role of *KCNMA1* in myopia progression is needed, particularly in ion channel activity which is one of the major functional pathways implicated, with an existing pool of several associated genes (*KCNQ5*, *KCNJ2*, and *CACNA1D*) [7].

As the first two susceptibility loci found to be associated with myopia [6, 8, 9, 12, 18, 57, 58], *GJD2* [59, 60] and *RASGRF1* [13, 17, 61] were significantly associated with HM in those with HM and MMD in the current study and previous studies. However, similar to previous work, *GJD2* [31] and *RASGRF1* [17, 31] were not specifically associated with MMD. *GJD2* [62–65] plays an essential role in synaptic transmission and processing of visual signals in photoreceptors and retinal cells [64, 66–69], and seems to be controlled by light exposure and dopamine [70], both of which have established roles in eye growth and myopia development [57, 71, 72]. *RASGRF1* [66, 68, 73] is involved in neuronal signal transduction pathways for retinal maintenance and function, and synaptic transmission of the photoreceptor responses [68]. Downregulated *RASGRF1* expression in mice models have resulted in impaired memory consolidation and learning [74], and deficiencies in photoreception and visual sensory processes [68].

We acknowledge that there may be limitations to our study. Our study is likely to be underpowered to detect associations between the candidate genetic variants and HM and MMD, due to the small sample size of MMD cases. The direction of effect in our study were similar to that in the previously reported GWAS for *KCNMA1* and *GJD2*,[11] thus indicating the lack of sufficient power in this study. As the prevalence of MMD in the population is low at 1–3% [2], it is logistically difficult to collect a sufficient number of cases with both MMD and genotyping information. It is unclear if the genetic variants reported in our findings are associated with HM, as the control group used in the first analysis should ideally be low and moderate myopes without MMD, instead of emmetropes without MMD. Therefore, these genetic variants are associated with HM in a specific population of those with both MMD and HM. We do not have individual data from each participating study on other factors that might be associated with MMD, for instance myopia duration. Although our study population has ethnic diversity, comprising individuals of European and Asian ancestries, the nonsignificant associations may be due to genetic heterogeneity across populations of varying ethnicity. It may be due to the multifactorial influences on this complex ocular disease as well. In addition, due to the nature of our candidate study, we focused on SNPs with strong prior evidence of association with myopia. In that respect, it may be unsurprising that these loci did not confer any significant effect over MMD, independent of their effect over HM. We did not conduct a GWAS and this study evaluated only the top SNPs for myopia, thus genetic variants that specifically affect MMD but have weak or no associations with myopia were not examined in this study. As only the top SNPs with stronger association with myopia were tested, we may have missed significant associations of untested SNPs with weaker associations with myopia present in the same locus as the top SNPs that were tested. Further verification and replication of our findings are required.

## Conclusions

In our study, we did not find any myopia-associated variant that was specifically associated with MMD. However, we report a significant association between HM in highly myopic subjects with MMD and the rs10824518 SNP in the *KCNMA1* locus in an international and multi-ethnic study. We also replicated and verified associations between HM in highly myopic subjects with MMD and the first gene associated with SE (*GJD2*). Further studies of larger sample sizes are required to elucidate susceptibility loci exclusive to MMD.

## Supporting information

S1 Text Membership of the CREAM Consortium. (DOCX)

S2 Text Study populations and acknowledgments. (DOCX)

S1 Appendix Meta-analysis on genetic association studies checklist. (DOCX)

S2 Appendix Plot of the effect on high myopia in highly myopic subjects with myopic macular degeneration for all 39 tested SNPs in the population cohorts in first case-control study. (DOCX)

S3 Appendix Plot of the effect on myopic macular degeneration in highly myopic subjects with myopic macular degeneration for all 37 tested SNPs in the population cohorts in second case-control study. (DOCX)
