# Correction: Genetic variants linked to myopic macular degeneration in persons with high myopia: CREAM Consortium

**DOI:** 10.1371/journal.pone.0223942

**Published:** 2019-10-10

**Authors:** Yee-Ling Wong, Pirro Hysi, Gemmy Cheung, Milly Tedja, Quan V. Hoang, Stuart W. J. Tompson, Kristina N. Whisenhunt, Virginie J. M. Verhoeven, Wanting Zhao, Moritz Hess, Chee-Wai Wong, Annette Kifley, Yoshikatsu Hosoda, Annechien E. G. Haarman, Susanne Hopf, Panagiotis Laspas, Sonoko Sensaki, Xueling Sim, Masahiro Miyake, Akitaka Tsujikawa, Ecosse Lamoureux, Kyoko Ohno-Matsui, Stefan Nickels, Paul Mitchell, Tien-Yin Wong, Jie Jin Wang, Christopher J. Hammond, Veluchamy A. Barathi, Ching-Yu Cheng, Kenji Yamashiro, Terri L. Young, Caroline C. W. Klaver, Seang-Mei Saw, The Consortium of Refractive Error, Myopia (CREAM)

The eighth author’s middle initials are missing. The correct name is: Virginie J. M. Verhoeven.
