# Supplementary material for: Genetic variants linked to myopic macular degeneration in persons with high myopia: CREAM Consortium
Source: PLoS One. 2019 Aug 15;14(8):e0220143. doi: 10.1371/journal.pone.0220143 (PMC6695159; doi:10.1371/journal.pone.0220143)
Supplement: S1 Text — (DOCX) [file pone.0220143.s001.docx]

**S1 Text. Membership of the CREAM Consortium**

Joan E. Bailey-Wilson^20^, Paul Nigel Baird^21^, Ginevra Biino^22^, Kathryn P. Burdon^23^, Harry Campbell^24^, Li Jia Chen^25^, Emily Y. Chew^26^, Jamie E. Craig^23^, Phillippa M. Cumberland^27^, Margaret M. Deangelis^28^, Cécile Delcourt^29^, Xiaohu Ding^30^, Cornelia M. van Duijn^9^, David M. Evans^31,32^, Qiao Fan^33^, Maurizio Fossarello^34^, Paul J. Foster^35^, Puya Gharahkhani^36^, Adriana I. Iglesias^8,9,11^, Jeremy A. Guggenheim^37^, Xiaobo Guo^30,38^, Toomas Haller^39^, Xikun Han^36^, Caroline Hayward^40^, Mingguang He^21,30^, Alex W. Hewitt^21,41,42^, Robert P. Igo Jr^43^, Sudha K. Iyengar^26,43,44^, Jost B. Jonas^44,45^, Mika Kähönen^46^, Jaakko Kaprio^47,48^, Anthony P. Khawaja^35,49^, Barbara E. K. Klein^10^, Ronald Klein^10^, Jonathan H. Lass^43,50^, Kris Lee^10^, Terho Lehtimäki^51,52^, Deryana Lewis^20^, Qing Li^53^, Shi-Ming Li^44^, Leo-Pekka Lyytikäinen^51,52^, Stuart MacGregor^36^, David A. Mackey^21,41,42^, Nicholas G. Martin^54^, Akira Meguro^55^, Andres Metspalu^39^, Candace Middlebrooks^20^, Nobuhisa Mizuki^55^, Anthony Musolf^20^, Konrad Oexle^56^, Chi Pui Pang^25^, Olavi Pärssinen^57,58^, Andrew D. Paterson^59^, Norbert Pfeiffer^60^, Ozren Polašek^61^, Jugnoo S. Rahi^27,35,62^,Olli Raitakari^63,64^, Igor Rudan^24^, Srujana Sahebjada^21^, Dwight Stambolian^65^, Claire L. Simpson^20,66^, E-Shyong Tai^2^, J. Willem L. Tideman^8,9^, Veronique Vitart^40^, Ningli Wang^44^, Juho Wedenoja^47,67^, Wen Bin Wei^68^, Ya Xing Wang^44^, Cathy Williams^69^, Katie M. Williams^4^, James F. Wilson^24,40^, Robert Wojciechowski^20,70,71^, Jason C. S. Yam^25^, Maurice K. H. Yap^72^, Seyhan Yazar^42^, Shea Ping Yip^73^ and Xiangtian Zhou^74^

^1^Singapore Eye Research Institute, Singapore National Eye Centre, Singapore.

^2^Saw Swee Hock School of Public Health, National University of Singapore, Singapore.

^3^R&D Vision Sciences AMERA, Essilor International, Singapore.

^4^Section of Academic Ophthalmology, School of Life Course Sciences, King’s College London, London, UK.

^5^Duke-NUS Medical School, Singapore.

^6^Yong Loo Lin School of Medicine, National University of Singapore and National University Health System, Singapore.

^7^Department of Ophthalmology, Columbia University Medical Center, New York, NY, USA.

^8^Department of Ophthalmology, Erasmus Medical Center, Rotterdam, The Netherlands.

^9^Department of Epidemiology, Erasmus Medical Center, Rotterdam, The Netherlands.

^10^Department of Ophthalmology and Visual Sciences, University of Wisconsin, Madison WI.

^11^Department of Clinical Genetics, Erasmus Medical Center, Rotterdam, The Netherlands.

^12^Institute for Medical Biostatistics, Epidemiology and Informatics, University Medical Center of the Johannes Gutenberg - University Mainz, Mainz, Germany.

^13^Institute of Medical Biometry and Statistics, Faculty of Medicine and Medical Center - University of Freiburg, Freiburg, Germany.

^14^Department of Ophthalmology, Centre for Vision Research, Westmead Institute for Medical Research, University of Sydney, Sydney, New South Wales, Australia.

^15^Department of Ophthalmology and Visual Sciences, University Graduate School of Medicine, Kyoto, Japan.

^16^Department of Ophthalmology, University Medical Center of the Johannes Gutenberg - University Mainz, Mainz, Germany.

^17^Department of Ophthalmology and Visual Science, Tokyo Medical and Dental University, Tokyo, Japan.

^18^Department of Ophthalmology, Otsu Red-Cross Hospital, Otsu, Japan.

^19^Department of Ophthalmology, Radboud University Medical Center, Nijmegen, The Netherlands.

^20^Computational and Statistical Genomics Branch, National Human Genome Research Institute, National Institutes of Health, Bethesda, MD, USA.

^21^Centre for Eye Research Australia, Ophthalmology, Department of Surgery, University of Melbourne, Royal Victorian Eye and Ear Hospital, Melbourne, Victoria, Australia.

^22^Institute of Molecular Genetics, National Research Council of Italy, Pavia, Italy.

^23^Department of Ophthalmology, Flinders University, Adelaide, South Australia, Australia.

^24^Centre for Global Health Research, Usher Institute for Population Health Sciences and Informatics, University of Edinburgh, Edinburgh, UK.

^25^Department of Ophthalmology and Visual Sciences, Chinese University of Hong Kong, Hong Kong Eye Hospital, Kowloon, Hong Kong.

^26^Department of Genetics, Case Western Reserve University, Cleveland, OH, USA.

^27^Great Ormond Street Institute of Child Health, University College London, London, UK.

^28^Department of Ophthalmology and Visual Sciences, John Moran Eye Center, University of Utah, Salt Lake City, UT, USA.

^29^Université de Bordeaux, Inserm, Bordeaux Population Health Research Center, team LEHA, UMR 1219, F-33000 Bordeaux, France.

^30^State Key Laboratory of Ophthalmology, Zhongshan Ophthalmic Center, Sun Yat-sen University, Guangzhou, China.

^31^Translational Research Institute, University of Queensland Diamantina Institute, Brisbane, Queensland, Australia.

^32^MRC Integrative Epidemiology Unit, University of Bristol, Bristol, UK.

^33^Centre for Quantitative Medicine, DUKE–National University of Singapore, Singapore, Singapore.

^34^University Hospital ‘San Giovanni di Dio’, Cagliari, Italy.

^35^NIHR Biomedical Research Centre, Moorfields Eye Hospital NHS Foundation Trust and UCL Institute of Ophthalmology, London, UK.

^36^Statistical Genetics, QIMR Berghofer Medical Research Institute, Brisbane, Queensland, Australia.

^37^School of Optometry & Vision Sciences, Cardiff University, Cardiff, UK.

^38^Department of Statistical Science, School of Mathematics, Sun Yat-Sen University, Guangzhou, China.

^39^Estonian Genome Center, University of Tartu, Tartu, Estonia.

^40^MRC Human Genetics Unit, MRC Institute of Genetics & Molecular Medicine, University of Edinburgh, Edinburgh, UK.

^41^Department of Ophthalmology, Menzies Institute of Medical Research, University of Tasmania, Hobart, Tasmania, Australia.

^42^Centre for Ophthalmology and Visual Science, Lions Eye Institute, University of Western Australia, Perth, Western Australia, Australia.

^43^Department of Population and Quantitative Health Sciences, Case Western Reserve University, Cleveland, OH, USA.

^44^Beijing Institute of Ophthalmology, Beijing Key Laboratory of Ophthalmology and Visual Sciences, Beijing Tongren Eye Center, Beijing Tongren Hospital, Capital Medical University, Beijing, China.

^45^Department of Ophthalmology, Medical Faculty Mannheim of the Ruprecht-Karls-University of Heidelberg, Mannheim, Germany.

^46^Department of Clinical Physiology, Tampere University Hospital and School of Medicine, University of Tampere, Tampere, Finland.

^47^Department of Public Health, University of Helsinki, Helsinki, Finland.

^48^Institute for Molecular Medicine Finland FIMM, HiLIFE Unit, University of Helsinki, Helsinki, Finland.

^49^Department of Public Health and Primary Care, University of Cambridge, Cambridge, UK.

^50^Department of Ophthalmology and Visual Sciences, Case Western Reserve University and University Hospitals Eye Institute, Cleveland, OH, USA.

^51^Department of Clinical Chemistry, Finnish Cardiovascular Research Center–Tampere, Faculty of Medicine and Life Sciences, University of Tampere, Tampere, Finland.

^52^Department of Clinical Chemistry, Fimlab Laboratories, University of Tampere, Tampere, Finland.

^53^National Human Genome Research Institute, National Institutes of Health, Baltimore, USA.

^54^Genetic Epidemiology, QIMR Berghofer Medical Research Institute, Brisbane, Queensland, Australia.

^55^Department of Ophthalmology, Yokohama City University School of Medicine, Yokohama, Japan.

^56^Institute of Neurogenomics, Helmholtz Zentrum München, German Research Centre for Environmental Health, Neuherberg, Germany.

^57^Department of Ophthalmology, Central Hospital of Central Finland, Jyväskylä, Finland.

^58^Gerontology Research Center, Faculty of Sport and Health Sciences, University of Jyväskylä, Jyväskylä, Finland.

^59^Program in Genetics and Genome Biology, Hospital for Sick Children and University of Toronto, Toronto, Ontario, Canada.

^60^Department of Ophthalmology, University Medical Center Mainz, Mainz, Germany.

^61^Faculty of Medicine, University of Split, Split, Croatia.

^62^Ulverscroft Vision Research Group, University College London, London, UK.

^63^Research Centre of Applied and Preventive Cardiovascular Medicine, University of Turku, Turku, Finland.

^64^Department of Clinical Physiology and Nuclear Medicine, Turku University Hospital, Turku, Finland.

^65^Department of Ophthalmology, University of Pennsylvania, Philadelphia, PA, USA.

^66^Department of Genetics, Genomics and Informatics, University of Tennessee Health Sciences Center, Memphis, TN, USA.

^67^Department of Ophthalmology, University of Helsinki and Helsinki University Hospital, Helsinki, Finland.

^68^Beijing Tongren Eye Center, Beijing Key Laboratory of Intraocular Tumor Diagnosis and Treatment, Beijing Ophthalmology & Visual Sciences Key Lab, Beijing Tongren Hospital, Capital Medical University, Beijing, China.

^69^Department of Population Health Sciences, Bristol Medical School, Bristol, UK.

^70^Department of Epidemiology and Medicine, Johns Hopkins Bloomberg School of Public Health, Baltimore, MD, USA.

^71^WilmerEye Institute, Johns Hopkins Medical Institutions, Baltimore, MD, USA.

^72^Centre for Myopia Research, School of Optometry, Hong Kong Polytechnic University, Hong Kong, Hong Kong.

^73^Department of Health Technology and Informatics, Hong Kong Polytechnic University, Hong Kong, Hong Kong.

^74^School of Ophthalmology and Optometry, Eye Hospital, Wenzhou Medical University, Wenzhou, China.
