# Supplementary material for: Genetic variants linked to myopic macular degeneration in persons with high myopia: CREAM Consortium
Source: PLoS One. 2019 Aug 15;14(8):e0220143. doi: 10.1371/journal.pone.0220143 (PMC6695159; doi:10.1371/journal.pone.0220143)
Supplement: S2 Text — (DOCX) [file pone.0220143.s002.docx]

**S2 Text. Study Populations and Acknowledgements**

*CREAM cohort*

CREAM (Consortium for Refractive Error and Myopia) was established in 2011 as a collaboration between studies with data on refractive error which had performed genome-wide association analysis based on SNP arrays. Details of each study cohort and their group-specific acknowledgements are provided below.

*Blue Mountains Eye Study (BMES)*

The Blue Mountains Eye Study (BMES) is a population-based cohort of a predominantly white population in west of Sydney, Australia. At baseline (1992-94), 3,654 permanent residents aged 49 years or older participated (participation rate of 82.4%). During 1997-99 (BMES II A), 2,335 participants (75.1% of survivors) returned for examinations after 5 years. During 1999-2000, 1,174 (85.2%) new participants took part in an Extension Study of the BMES (BMES IIB). BMES cross-section II thus includes BMES IIA (66.5%) and BMES IIB (33.5%) participants (n=3,509). From the BMES cross section II who had blood samples collected, DNA was extracted for 3,189 (90.1 %) participants. Over 98% of BMES participants were European ancestry. All BMES examinations were approved by the Human Ethics Committees of the Western Sydney Area Health Service and University of Sydney. Signed informed consent was obtained from participants at each examination.

*Acknowledgements BMES:*

BMES was supported by the Australian National Health & Medical Research Council (NH&MRC), Canberra Australia (974159, 211069, 457349, 512423, 475604, 529912); the Centre for Clinical Research Excellence in Translational Clinical Research in Eye Diseases; NH&MRC research fellowships (358702, 632909 to J.J.W, 1028444, 1138585 to P.N.B.); and the Wellcome Trust, UK as part of Wellcome Trust Case Control Consortium 2 (A. Viswanathan, P. McGuffin, P. Mitchell, F. Topouzis, P. Foster) for genotyping costs of the entire BMES population (085475B08Z, 08547508Z, 076113). The Centre for Eye Research Australia receives Operational Infrastructure Support from the Victorian government. BMES acknowledges Jie Jin Wang and Elena Rochtchina from the Centre for Vision Research, Department of Ophthalmology and Westmead Millennium Institute University of Sydney (NSW Australia); John Attia, Rodney Scott, Elizabeth G. Holliday from the University of Newcastle (Newcastle, NSW Australia); Jing Xie from the Centre for Eye Research Australia, University of Melbourne; Michael T. Inouye, Medical Systems Biology, Department of Pathology & Department of Microbiology & Immunology, University of Melbourne (Victoria, Australia); Ananth Viswanathan, Moorfields Eye Hospital (London, UK); Paul J. Foster, NIHR Biomedical Research Centre for Ophthalmology, UCL Institute of Ophthalmology & Moorfields Eye Hospital (London); Peter McGuffin, MRC Social Genetic and Developmental Psychiatry Research Centre, Institute of Psychiatry, King's College (London, United Kingdom); Fotis Topouzis, Department of Ophthalmology, School of Medicine, Aristotle University of Thessaloniki, AHEPA Hospital (Thessaloniki, Greece); Xueling Sim, National University of Singapore; members of the Wellcome Trust Case Control Consortium 2.

*Gutenberg Health Study (GHS1, GHS2)*

The Gutenberg Health Study is a population-based, prospective, observational cohort study in midwestern Germany that includes consecutive follow-ups every five years. The primary study aim is to evaluate and improve cardiovascular risk stratification and the general health status of the population. The baseline examination included a total of 15,010 participants aged 35 to 74 years and took place from 2007 to 2012. The participants were randomly drawn and equally stratified for sex, residence (urban or rural) and for each decade of age. Exclusion criteria were the following: insufficient knowledge of German and physical or mental inability to participate in the examinations in the study center. The ophthalmic examination was based on standard operating procedures and included without limitation autorefraction and visual acuity testing (Humphrey® Automated Refractor/Keratometer (HARK) 599™, Carl Zeiss Meditec AG, Jena, Germany). The study protocol and study documents were approved by the local ethics committee of the Medical Chamber of Rhineland-Palatinate, Germany (reference no. 837.020.07; original vote: 22.3.2007, latest update: 20.10.2015). According to the tenets of the Declaration of Helsinki, written informed consent was obtained from all participants prior to their entry into the study.

*Acknowledgements Gutenberg Health Study (GHS1, GHS2):*

The Gutenberg Health Study is funded through the government of Rhineland-Palatinate (“Stiftung Rheinland-Pfalz für Innovation“, contract AZ 961-386261/733), the research programs “Wissen schafft Zukunft” and “Center for Translational Vascular Biology (CTVB)” of the Johannes Gutenberg-University of Mainz, and its contracts with Boehringer Ingelheim and PHILIPS Medical Systems, including unrestricted grants for the Gutenberg Health Study. We thank all study participants for their willingness to provide data for this research project, and we are indebted to all coworkers for their enthusiastic commitment.

*Rotterdam Study (RS1, RS2, RS3)*

The Rotterdam Study is a prospective population-based cohort study in the elderly living in Ommoord, a suburb of Rotterdam, the Netherlands. Details of the study are described elsewhere. In brief, the Rotterdam Study consists of 3 independent cohorts: RS1, RS2, and RS3. For the current analysis, 5,328 residents aged 55 years and older were included from RS1, 2,009 participants aged 55 and older from RS2, and 1,970 aged 45 and older from RS 3. 99% of subjects were of European ancestry. Participants underwent multiple physical examinations with regular intervals from 1991 to present, including a nondilated automated measurement of refractive error using a Topcon RM-A2000 autorefractor. All measurements in RS-1–3 were conducted after the Medical Ethics Committee of the Erasmus University had approved the study protocols and all participants had given a written informed consent in accordance with the Declaration of Helsinki.

*Acknowledgements Rotterdam Study:*

We would like to acknowledge our graders Corina Brussee and Ada Hooghart. The Rotterdam Study I-III were supported by European Research Council (ERC) under the European Union's Horizon 2020 research and innovation programme (grant 648268), Netherlands Organisation for Scientific Research (NWO, grant 91815655), Erasmus Medical Center and Erasmus University, Rotterdam, The Netherlands; Netherlands Organization for Health Research and Development (ZonMw); UitZicht; Netherlands Organisation for Scientific Research (NWO Veni 91617076 to V.J.M.V.); the Research Institute for Diseases in the Elderly; the Ministry of Education, Culture and Science; the Ministry for Health, Welfare and Sports; the European Commission (DG XII); the Municipality of Rotterdam; the Netherlands Genomics Initiative/NWO; Center for Medical Systems Biology of NGI; Lijf en Leven; Henkes Stichting; Stichting Nederlands Oogheelkundig Onderzoek; Swart van Essen; Bevordering van Volkskracht; Blindenhulp; Landelijke Stichting voor Blinden en Slechtzienden; Rotterdamse Stichting Blindenbelangen; Oogfonds; Algemene Nederlandse Vereniging ter Voorkoming van Blindheid; Stichting MaculaFonds; Royal Netherlands Academy of Arts and Sciences, Combined Ophthalmic Research Rotterdam; Rotterdamse Oogheelkundig Onderzoek Stichting; Erasmus MC Vriendenfonds, Topcon Europe; Novartis.

*Nagahama*

Nagahama Prospective Cohort for Comprehensive Human Bioscience (the Nagahama Study) is a community-based cohort consisted of 9,804 healthy Japanese volunteers recruited between 2008 and 2010 from the general population of Nagahama City in Japan. Community residents from 30–74 years of age, living independently and without physical impairment or dysfunction were eligible. The Kyoto University Graduate School and Faculty of Medicine Ethics Committee and the Nagahama Municipal Review Board of Personal Information Protection approved the study protocol and procedures used to obtain informed consent. All the study procedures adhered to the tenets of the Declaration of Helsinki. All participants were fully informed about the purpose and procedures of the study, and written consent was obtained from each subject.

*Acknowledgements Nagahama:*

Nagaham Study was financially supported by Comprehensive Research on Aging and Health Science Research Grants for Dementia R&D from Japan Agency for Medical Research and Development (AMED) and the Centre of Innovation Program, the Global University Project from Japan Science and Technology Agency.

*Singapore Studies*

All Singapore studies adhere to the Declaration of Helsinki. Ethics approvals have been obtained from the Institutional Review Boards of the Singapore Eye Research Institute, Singapore General hospital, National University of Singapore and National Healthcare Group, Singapore. In all cohorts, participants provided written, informed consent at the recruitment into the studies.

*Singapore Malay Eye Study (SiMES)*

SiMES is a population-based prevalence survey of Malay adults aged 40 to 79 years living in Singapore that was conducted between August of 2004 and June of 2006. From a Ministry of Home Affairs, random sample of 16,069 Malay adults in the Southwestern area, an age-stratified random sampling strategy was used in selecting 1,400 from each decade from age 40 years onward (40–49, 50–59, 60–69, and 70–79 years). The 4,168 eligible participants from the sampling frame, while 3280 (78.7%) participated.

*Acknowledgements Singapore Malay Eye Study (SiMES):*

See “Acknowledgements Singapore Studies”

*Singapore Indian Eye Study (SINDI)*

SINDI is a population-based survey of major eye diseases in ethnic Indians aged 40 to 80 years living in the South-Western part of Singapore and was conducted from August 2007 to December 2009. In brief, 4,497 Indian adults were eligible and 3,400 participated.

*Acknowledgements Singapore Indian Eye Study (SINDI):*

See “Acknowledgements Singapore Studies”

*Singapore Chinese Eye Study (SCES)*

Similar to SINDI, the Singapore Chinese Eye Study (SCES) is a population-based cross-sectional study of eye diseases in Chinese adults 40 years of age or older residing in the southwestern part of Singapore. The methodology of the SCES study has been described in details previously. Between 2009 and 2011, 3,353 (72.8%) of 4,605 eligible individuals underwent a comprehensive ophthalmologic examination, using the same protocol as SINDI.

*Acknowledgements Singapore Chinese Eye Study (SCES):*

see ”Acknowledgements Singapore Studies”

*Acknowledgements Singapore Studies:*

The Singapore studies (SiMES, SINDI, SCES) were supported by the National Medical Research Council (grants: 0796/2003, IRG07nov013, IRG09nov014, STaR/0003/2008; CG/SERI/2010), Biomedical Research Council (grants: 08/1/35/19/550, 09/1/35/19/616), Economic Development Board-Essilor Post-graduate Programme (S14-1103-IPP), Singapore. The Singapore Tissue Network and the Genome Institute of Singapore, Agency for Science, Technology and Research, Singapore provided services. National supercomputing centre (NSCC) provided high performance computing resources to support GWAS analysis.

*Acknowledgements expression study Young TL et al:*

This study was funded by NIH/NEI R01 EY014685, Research To Prevent Blindness, Inc., University of Wisconsin Centennial Scholars Fund.
