# Supplementary material for: Genetic variants linked to myopic macular degeneration in persons with high myopia: CREAM Consortium
Source: PLoS One. 2019 Aug 15;14(8):e0220143. doi: 10.1371/journal.pone.0220143 (PMC6695159; doi:10.1371/journal.pone.0220143)
Supplement: S1 Appendix — (DOCX) [file pone.0220143.s003.docx]

**Meta-analysis on Genetic Association Studies Checklist | PLOS ONE**

|  | Item | Section name and paragraph number within manuscript |
| --- | --- | --- |
|  | **Introduction** |  |
| 1 | Provide a detailed justification for the polymorphism studied; if a single polymorphism was analyzed, give details as to why others were not included in the meta-analysis. | Introduction (Lines 95-99);  Methods (Lines 162-183) |
| 2 | Provide a detailed justification for the population(s) and clinical condition studied. | Methods (Lines 120-160) |
|  | **Methods** |  |
| 3 | Provide full details of the search strategy employed; outline the full electronic search strategy –specific combination of keywords and any limits applied- for at least one database. Specify whether synonyms of polymorphisms/genes (e.g. SNP number) were searched. | Methods (Lines 164-168) |
| 4 | Report full details on the inclusion and exclusion criteria applied for selecting studies.  *Please list the excluded articles and the reasons for exclusion of each article in a supplementary file.* | Methods (Lines 121-126) |
| 5 | Provide details on how the quality of the studies included in the analyses was assessed. | Methods (Lines 185-194) |
| 6 | Describe steps taken to contact study authors to identify additional studies and to request missing data. | Various studies from the CREAM consortium that fit the inclusion/exclusion criteria were identified, and data was requested correspondents for each participating study. |
| 7 | Describe how environmental effects were adjusted for, if this adjustment was not conducted, outline the reasons for this. | The adjustment for environmental effects for MMD were not conducted, as data on environmental factors were not available in most studies. |
| 8 | Describe the methods of handling heterogeneity/between-study variance. | Methods (Lines 223-233) |
| 9 | Describe how the Hardy-Weinberg equilibrium and linkage disequilibrium were assessed. | Methods (Lines 185-194) |
| 10 | Describe and justify the choice of model for the analyses (per-allele vs per-genotype vs genetic model-free, random effects vs fixed effects). | Methods (Lines 225-227) |
| 11 | Describe whether a sensitivity analysis has been completed. | A sensitivity analysis has not been completed. |
| 12 | Describe whether an assessment of the effects of population stratification has been conducted. | An assessment of the effects of population stratification has not been conducted. |
| 13 | Describe whether study-specific results have been assessed and if so the reasons for this (e.g. forest plot). | Study-specific results have been assessed, but only the combined effects in meta-analyses were presented. |
|  | **Results** |  |
| 14 | Include flow diagram for the studies included in the meta-analysis as the first figure for the manuscript | There is no flow diagram, but Table 1 describes the studies included in the meta-analyses. |
| 15 | Report details on allele/genotype prevalence. | Results (Lines 236-240) |
| 16 | Report the effect size estimates and p values for each analysis. | Tables 2 and 3 |
|  | **Discussion** |  |
| 17 | Discuss the limitations of the meta-analysis, including genotyping errors/bias and publication bias. | Discussion (Lines 385-410) |
| 18 | If the meta-analysis identifies an association within a subgroup of the population studied but not another, discuss the implications of these results, and if applicable the possibility of subgroup-specific publication bias. | Not applicable. |
| 19 | Discuss the suitability of the sample size employed to the research question and the power of the study. | Discussion (Lines 385-391): This study is likely to be underpowered, due to the small sample size of MMD cases. There is logistic difficulty in collecting sufficient numbers of cases with both MMD and genotyping data. |
