# Supplementary material for: Genetic variants linked to myopic macular degeneration in persons with high myopia: CREAM Consortium
Source: PLoS One. 2019 Aug 15;14(8):e0220143. doi: 10.1371/journal.pone.0220143 (PMC6695159; doi:10.1371/journal.pone.0220143)
Supplement: S3 Appendix — (DOCX) [file pone.0220143.s005.docx]

**S2 Appendix. Plot of the effect on myopic macular degeneration in highly myopic subjects with myopic macular degeneration for all 37 tested SNPs in the population cohorts in second case-control study.**

| 1:164213686 | 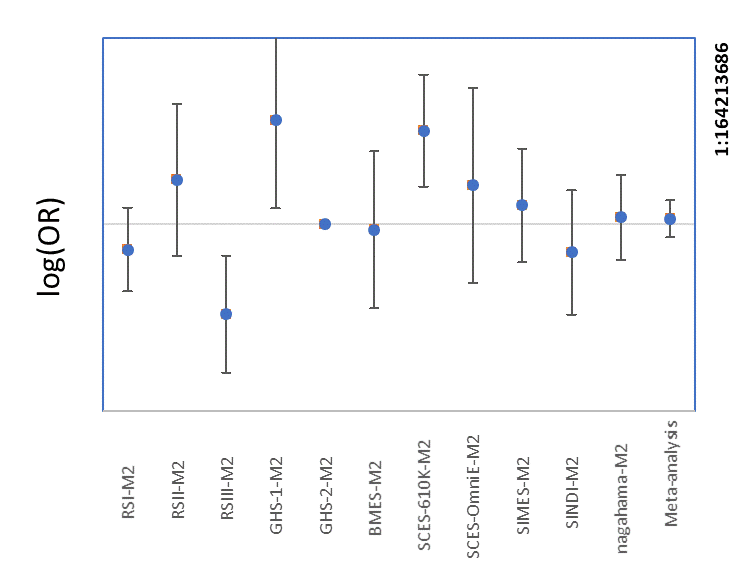 |
| --- | --- |
| 1:200336075 | 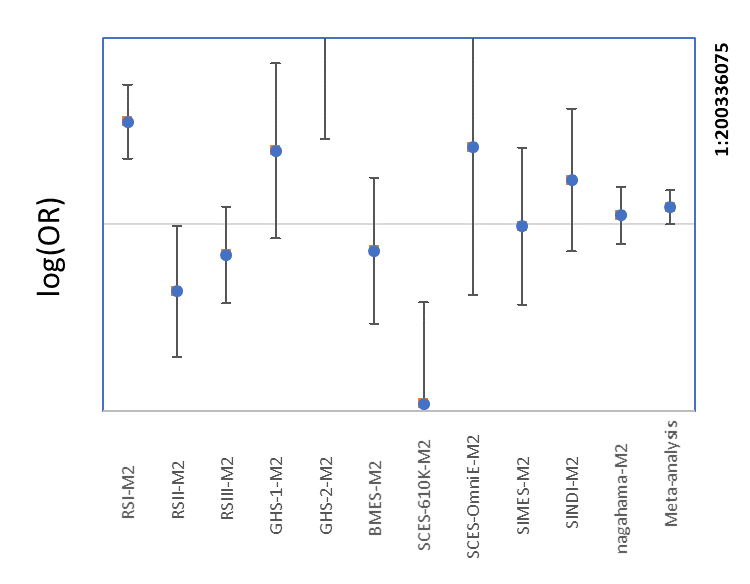 |
| 1:61341632 | 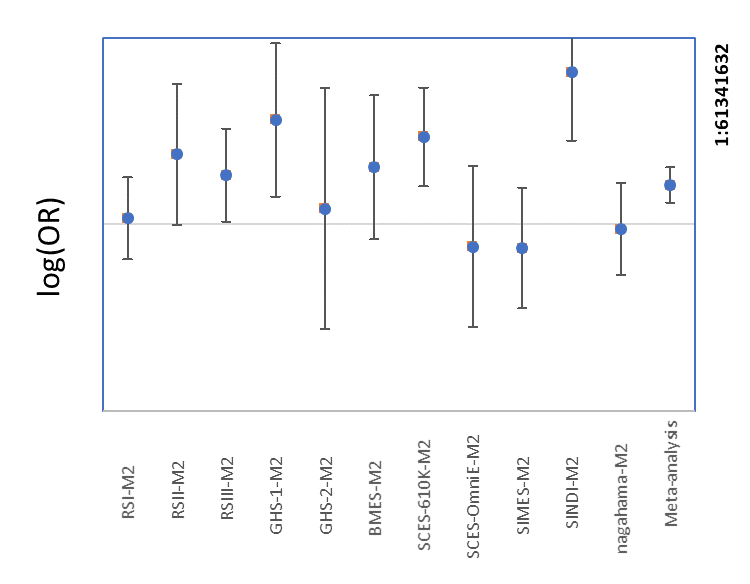 |
| 10:60339098 | 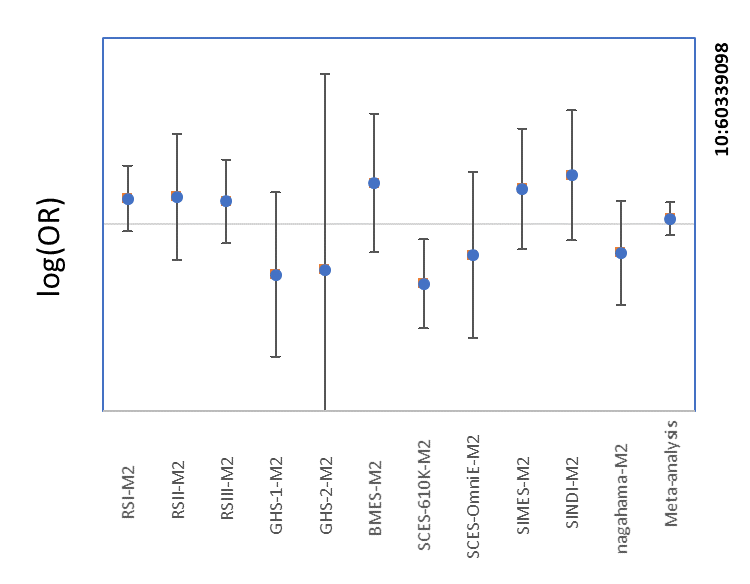 |
| 10:79063542 | 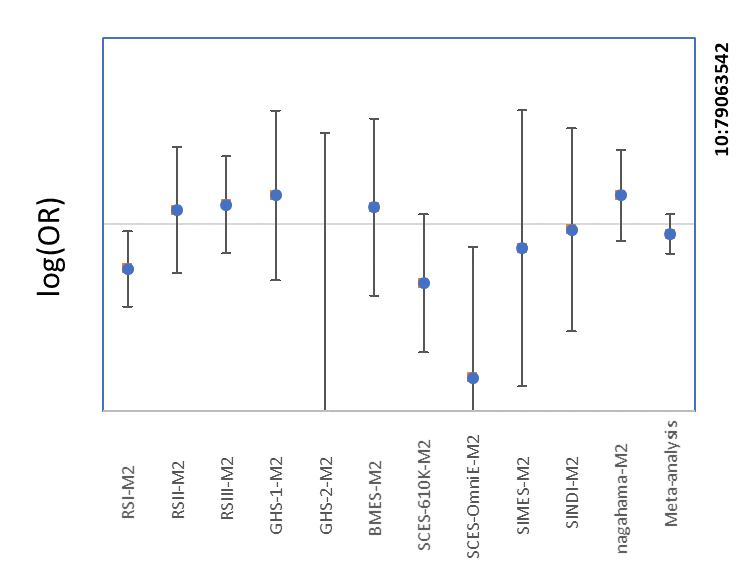 |
| 10:86015573 | 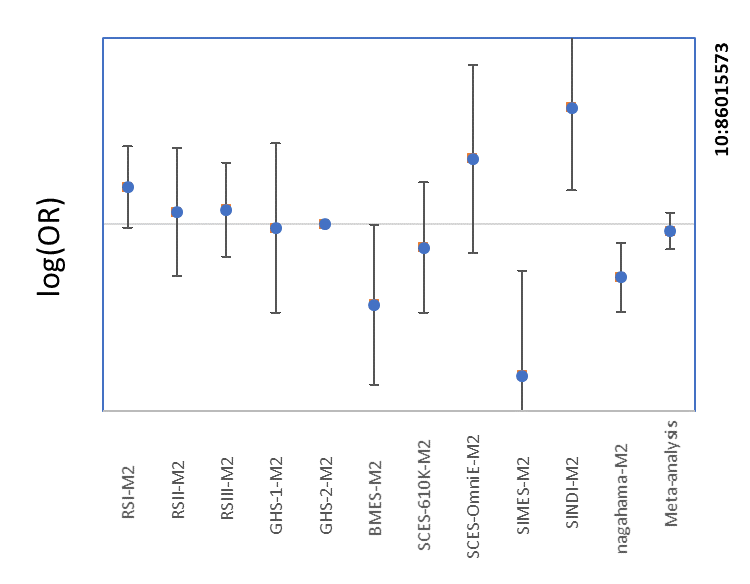 |
| 11:131931531 | 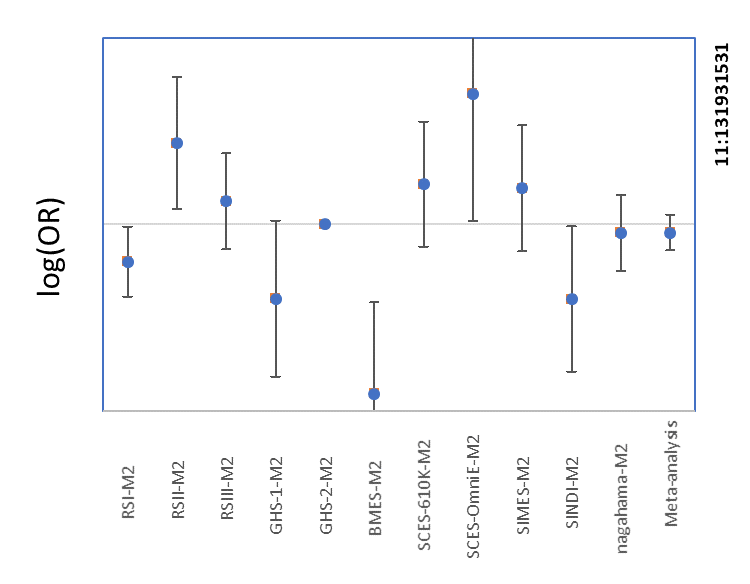 |
| 11:18751041 | 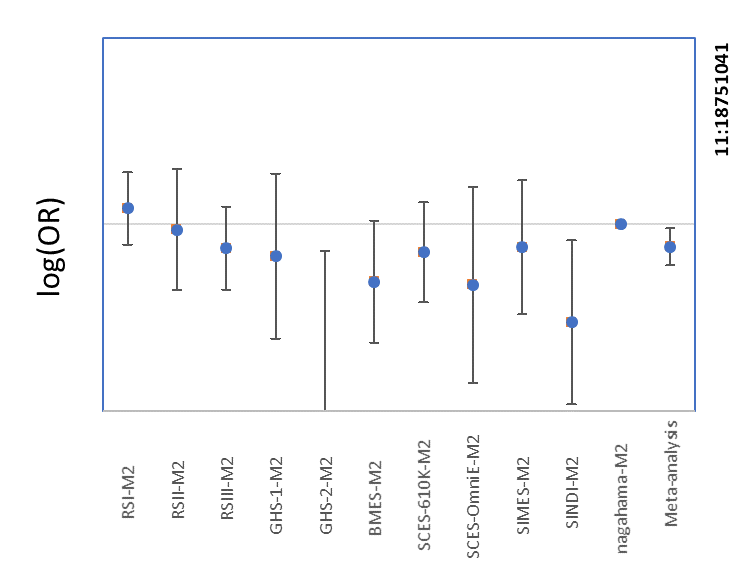 |
| 11:40149300 | 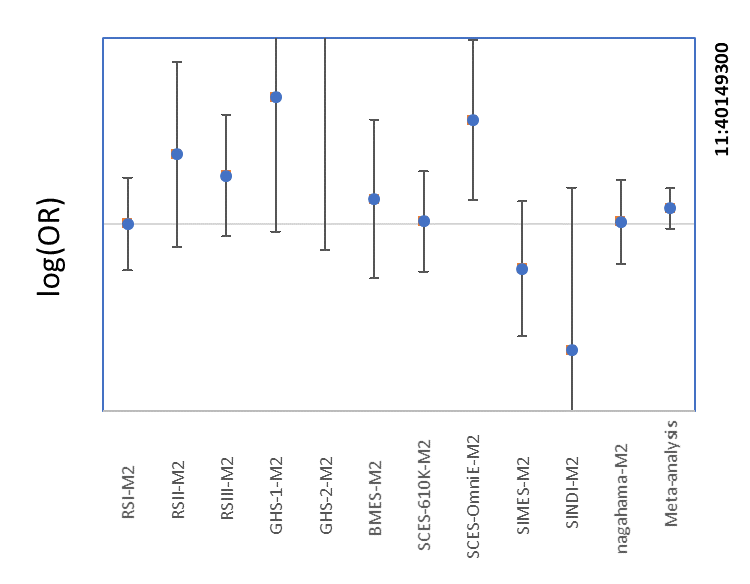 |
| 11:84634790 | 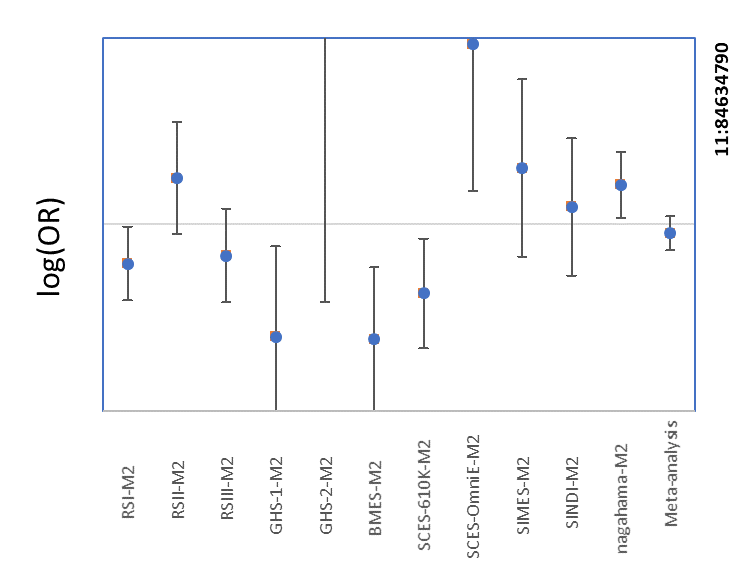 |
| 12:9313304 | 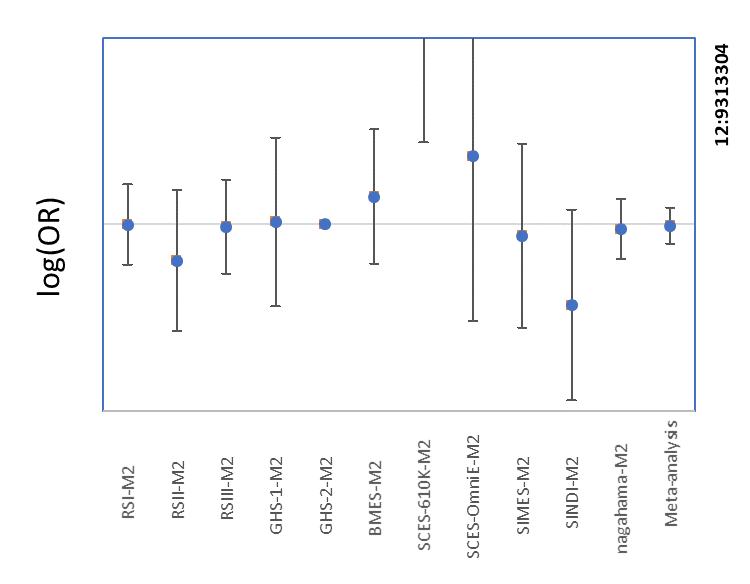 |
| 13:100689354 | 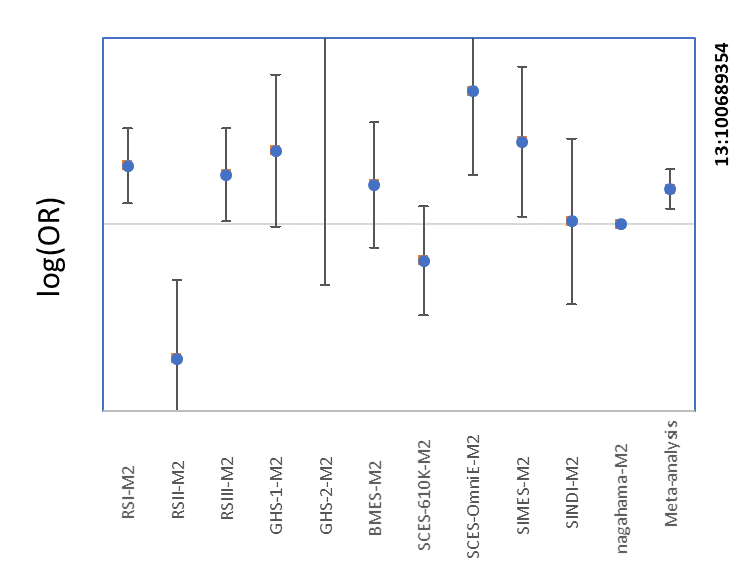 |
| 14:54552428 | 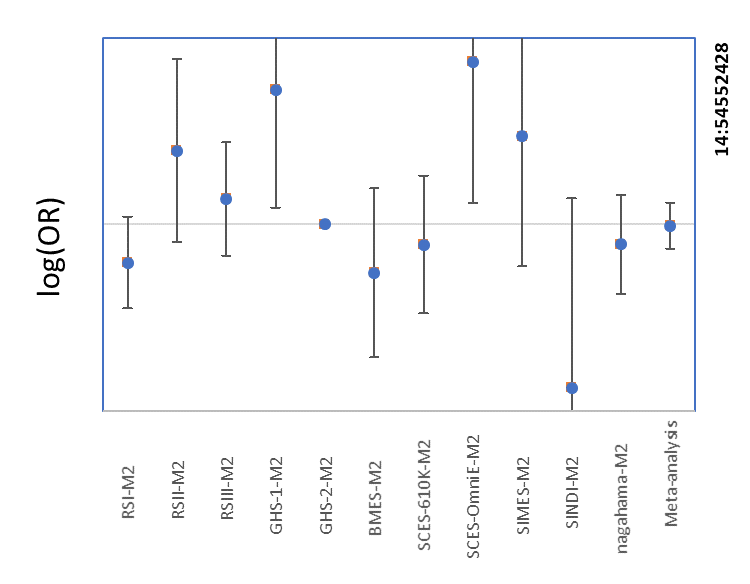 |
| 15:35005886 | 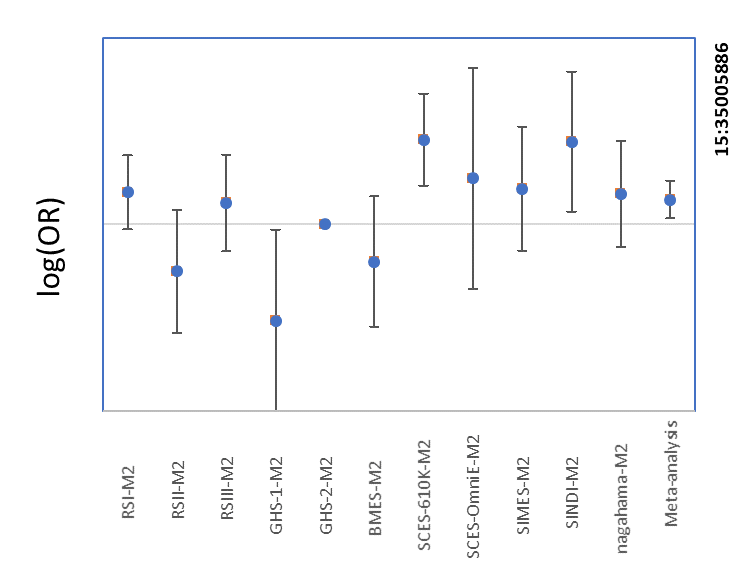 |
| 15:63571234 | 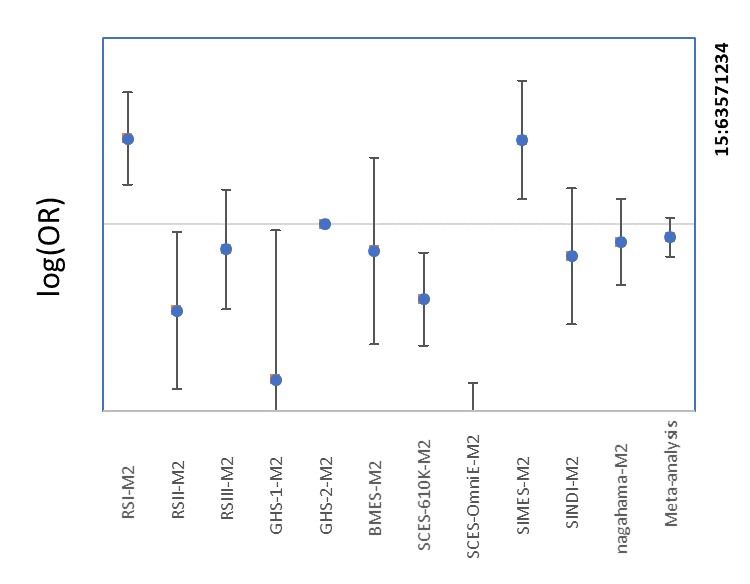 |
| 15:79378821 | 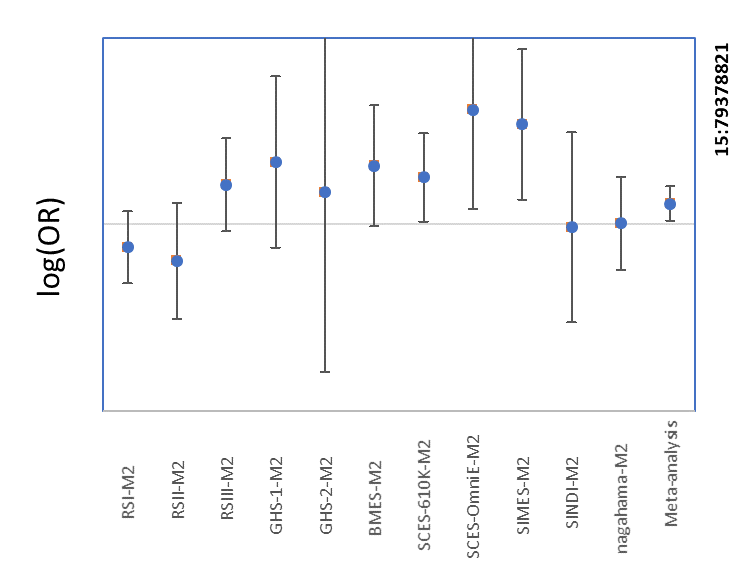 |
| 17:11407259 | 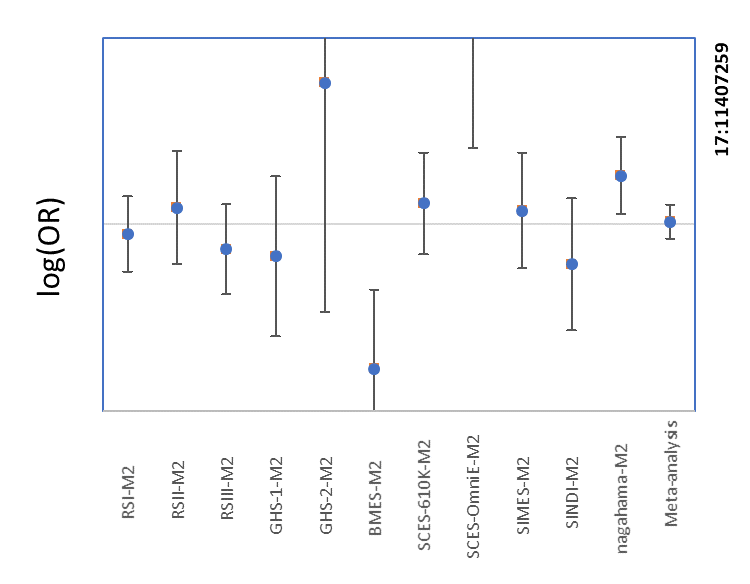 |
| 17:7429321 | 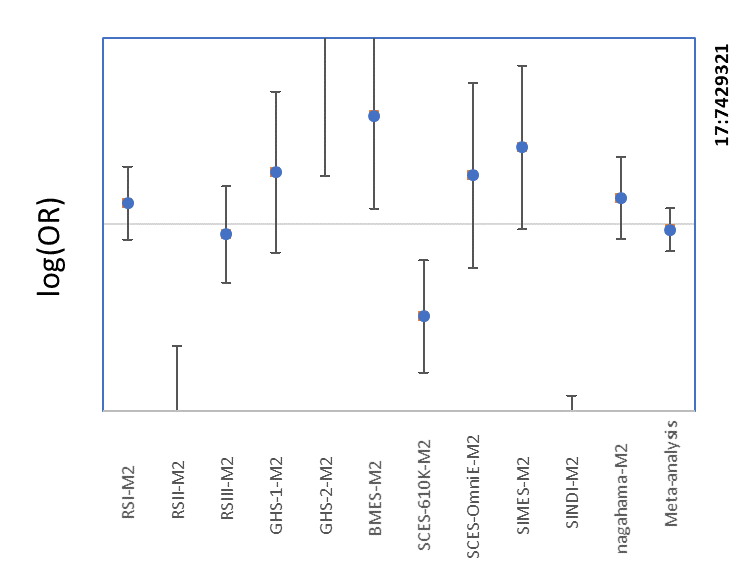 |
| 18:47391025 | 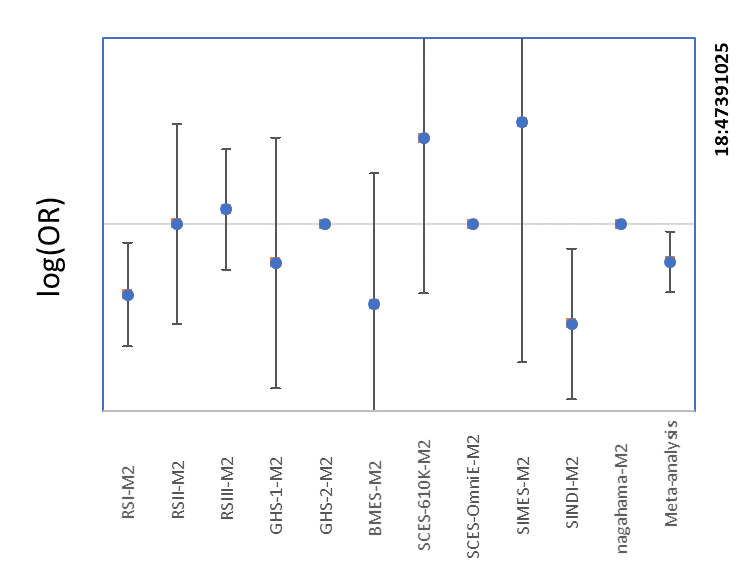 |
| 2:146888708 | 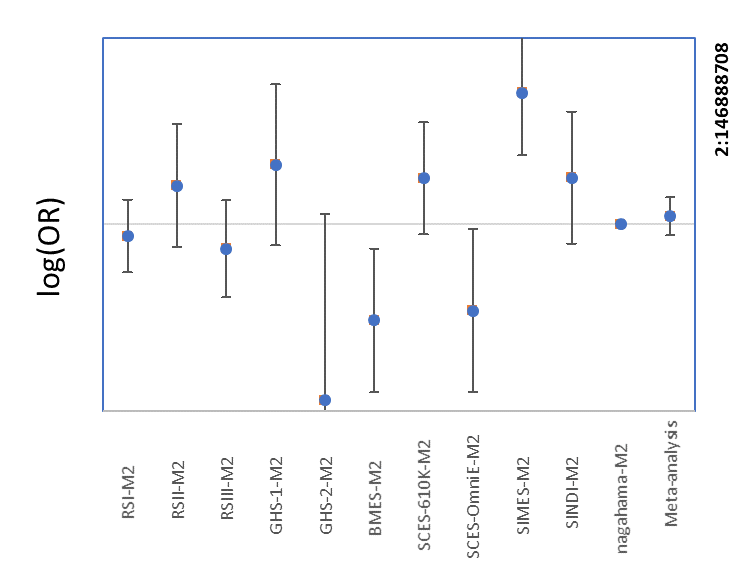 |
| 2:157358750 | 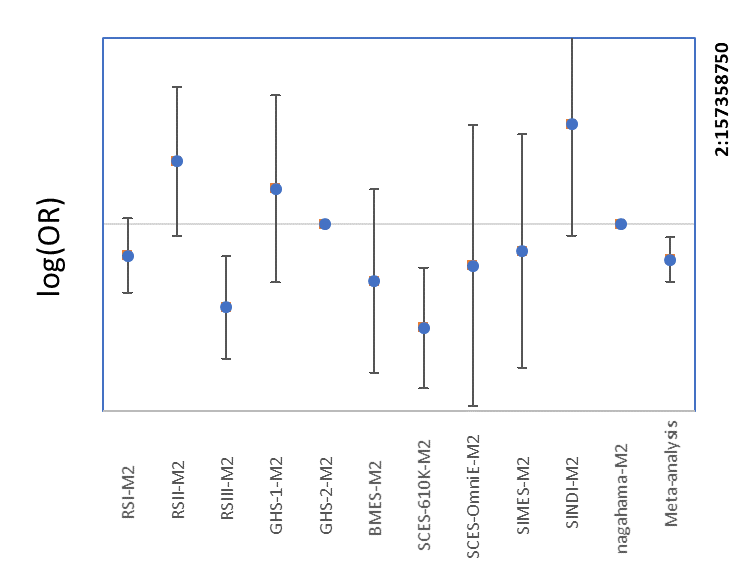 |
| 2:172851936 | 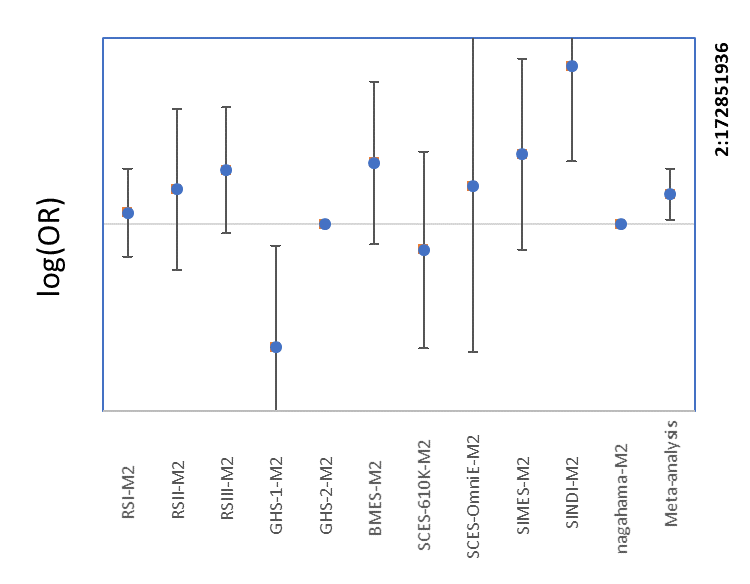 |
| 2:233385396 | 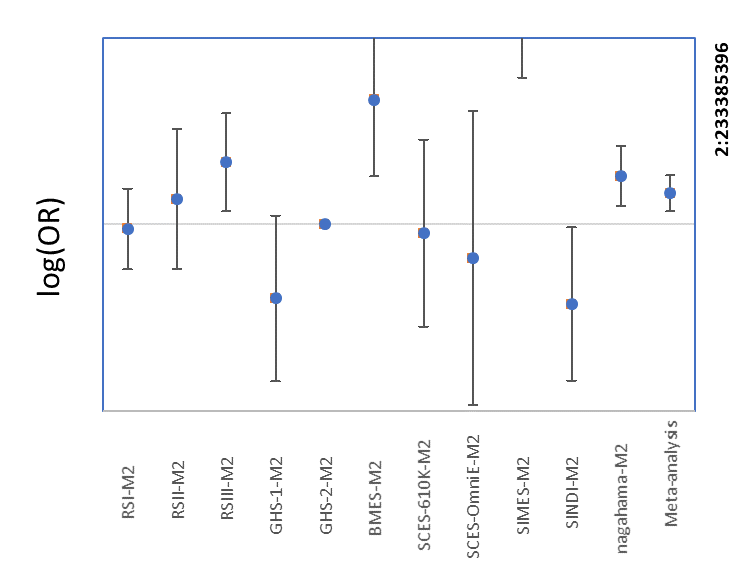 |
| 2:45152748 | 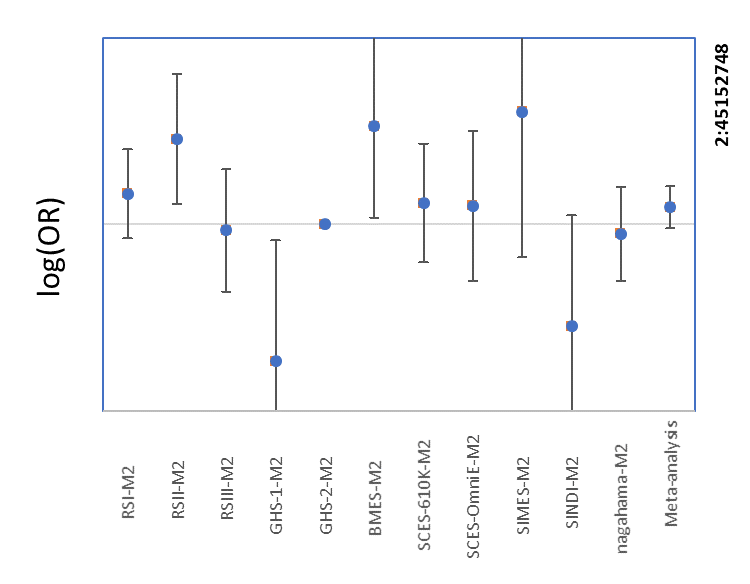 |
| 21:47371947 | 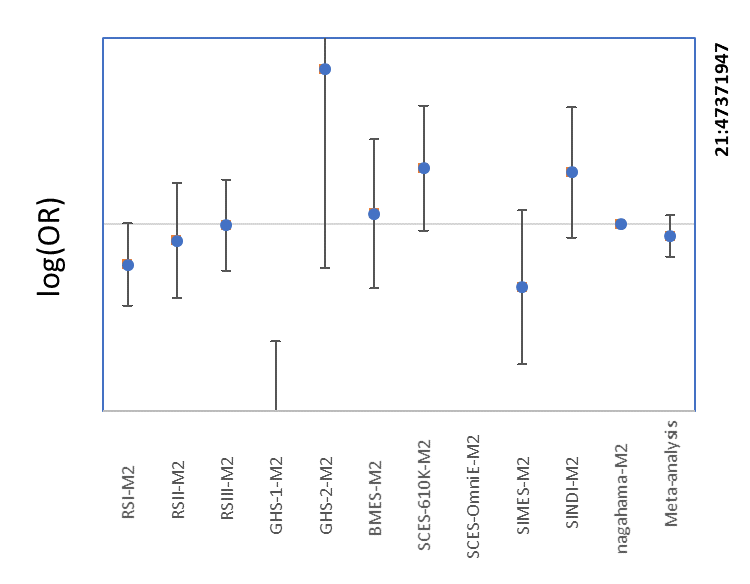 |
| 3:141076084 | 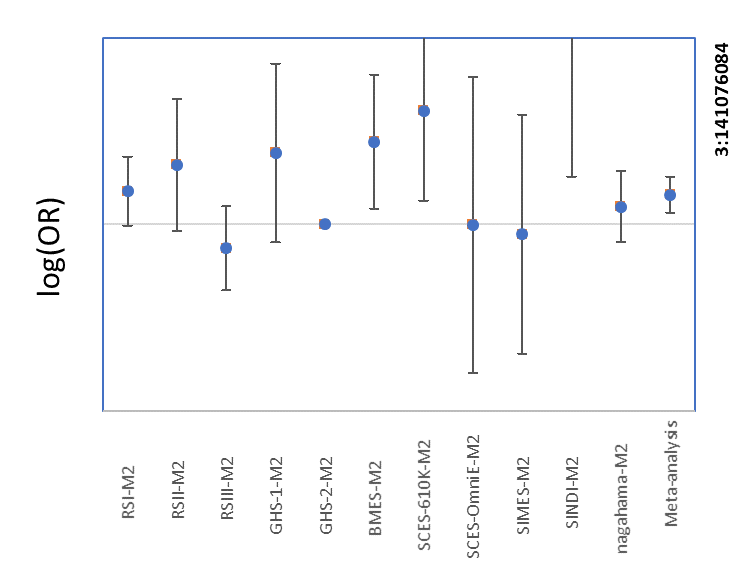 |
| 3:24268677 | 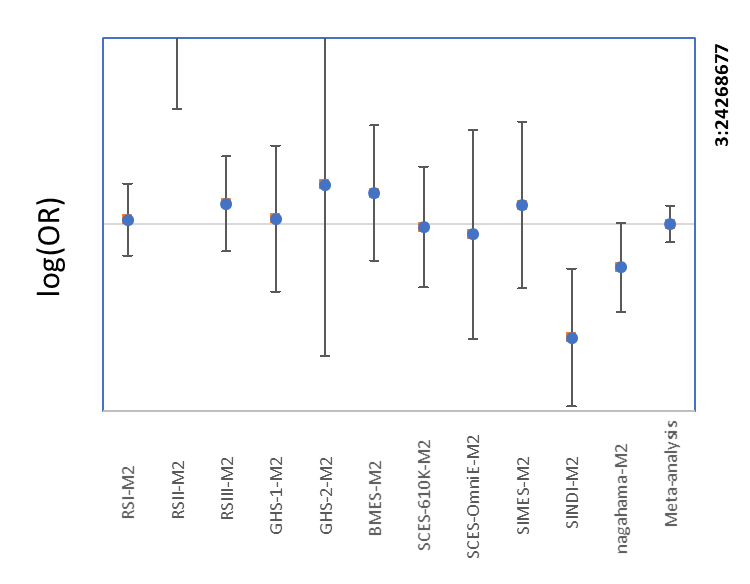 |
| 4:80508788 | 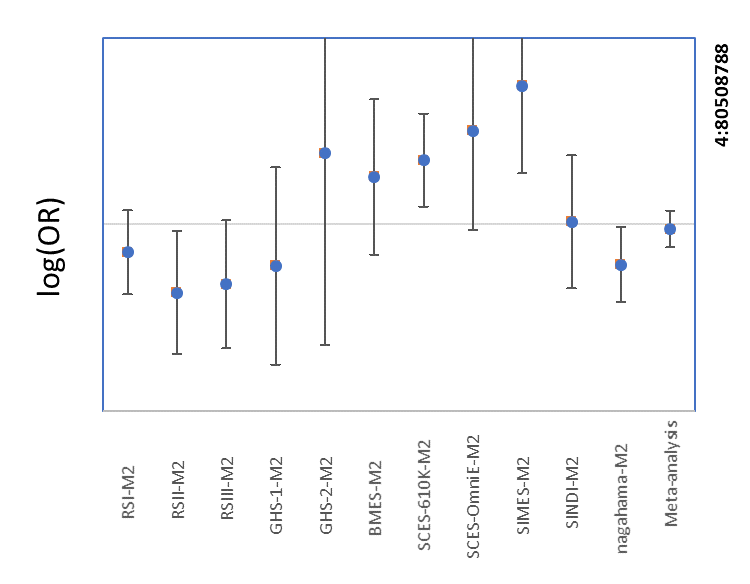 |
| 4:81959966 | 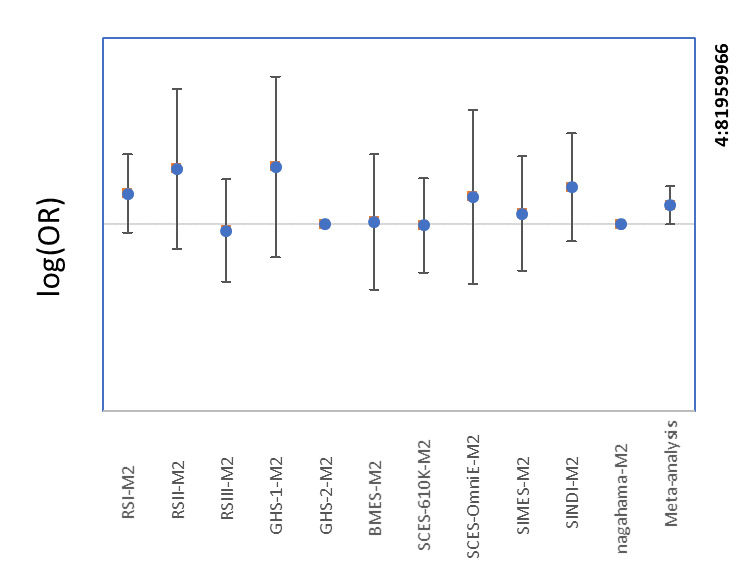 |
| 6:116446576 | 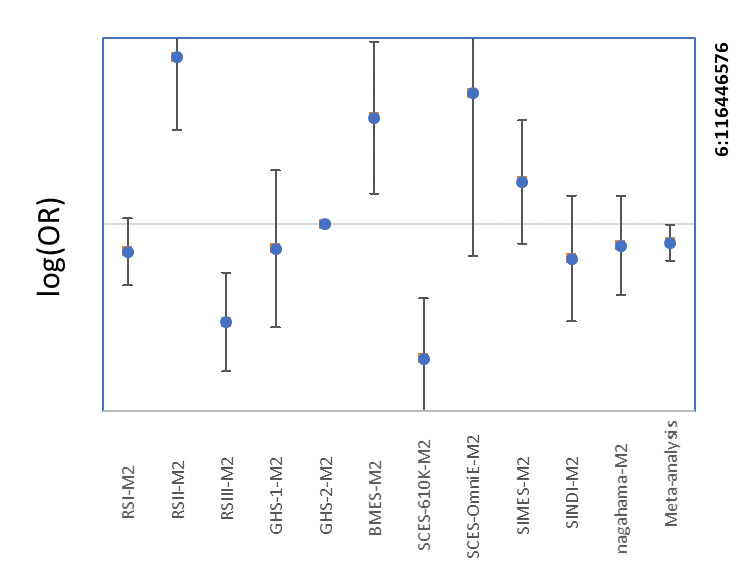 |
| 6:28270584 | 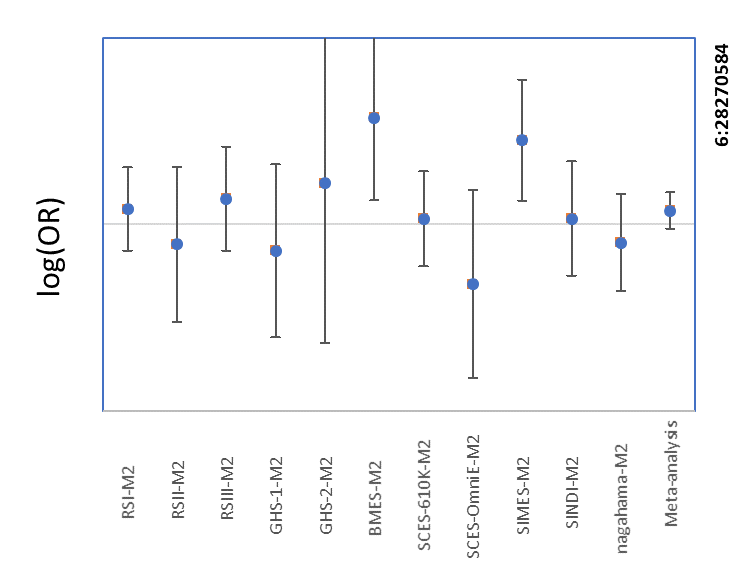 |
| 6:50809720 | 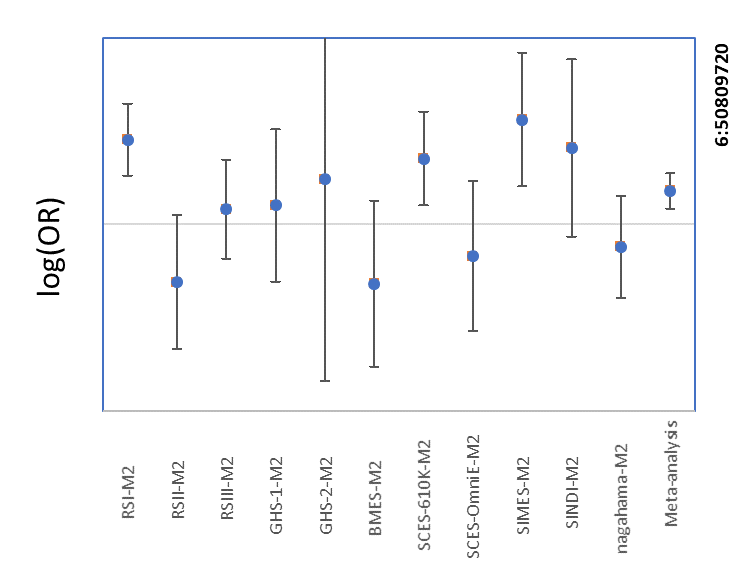 |
| 6:73643289 | 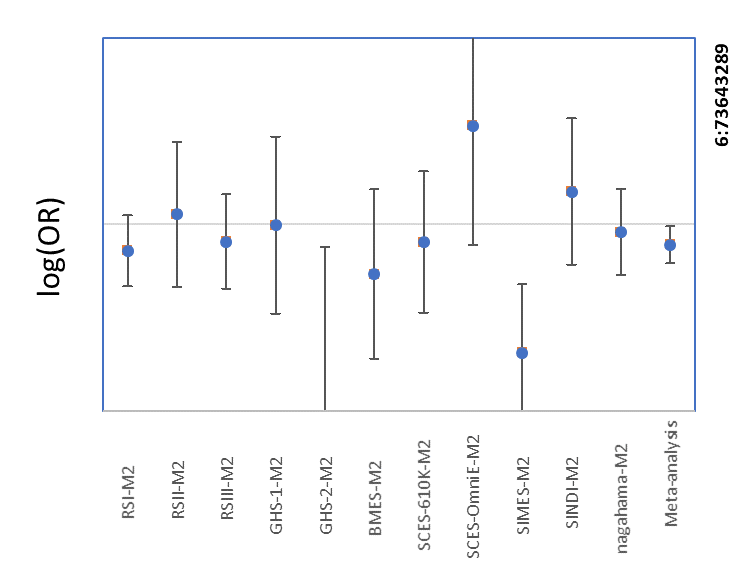 |
| 8:40723970 | 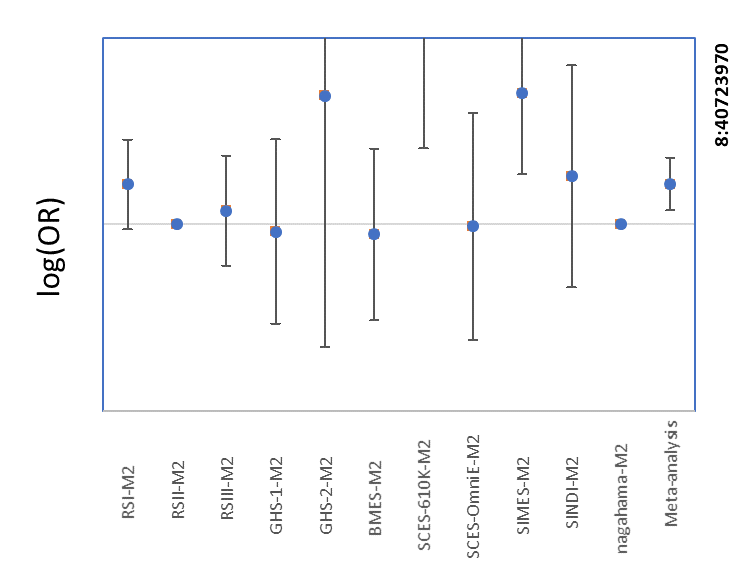 |
| 8:60178721 | 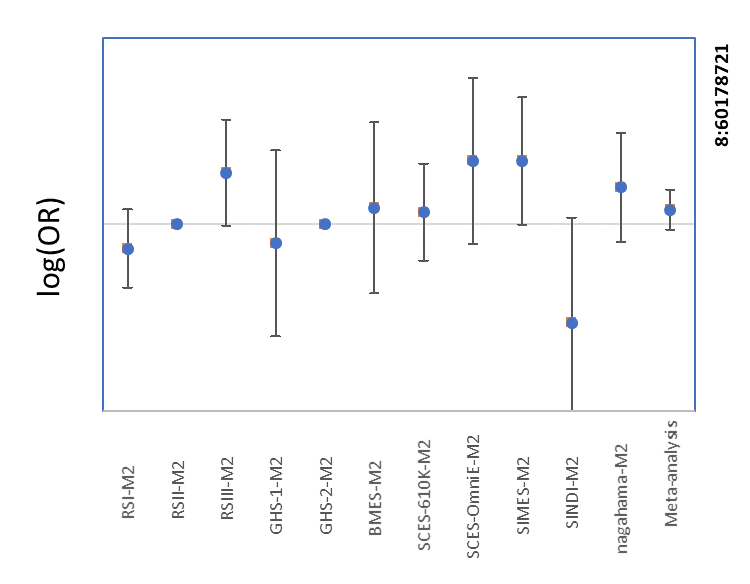 |
| 9:18362865 | 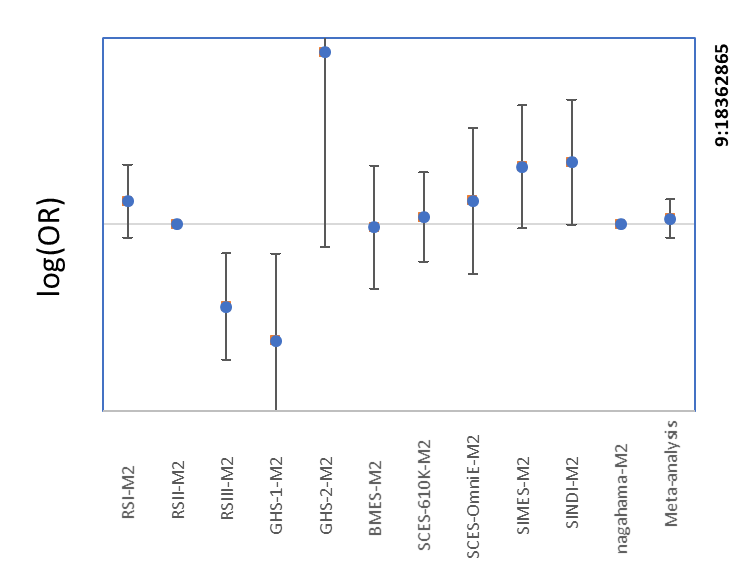 |
